# Biopriming with *Schizophyllum commune* Polysaccharides Modulates Antioxidant and Biochemical Responses in *Pisum sativum* L. Seedlings

**DOI:** 10.3390/antiox15091145

**Published:** 2026-09-09

**Authors:** Jovana Mišković, Milena Rašeta, Gordana Gojgić-Cvijović, Marko Radenković, Nenad Krsmanović, Nemanja Živanović, Gordana Ćirić-Marjanović, Maja Milojević-Rakić, Gordana Tamindžić, Maja Karaman

**Affiliations:** 1 ProFungi Laboratory, Department of Biology and Ecology, Faculty of Sciences, University of Novi Sad, Trg Dositeja Obradovića 2, 21000 Novi Sad, Serbia; 2 Department of Chemistry, Biochemistry and Environmental Protection, Faculty of Sciences, University of Novi Sad, Trg Dositeja Obradovića 3, 21000 Novi Sad, Serbia; 3 Department of Chemistry, Institute of Chemistry, Technology and Metallurgy, University of Belgrade, Njegoševa 12, 11001 Belgrade, Serbia; 4 BioSense Institute, University of Novi Sad, Dr Zorana Đinđića 1, 21000 Novi Sad, Serbia; 5 Mycotopia LLC, Research and Development in Sciences, Lekina 37, 22232 Divoš, Serbia; 6 Faculty of Physical Chemistry, University of Belgrade, Studentski Trg 12–16, 11158 Belgrade, Serbia; 7 Institute of Field and Vegetable Crops, National Institute of the Republic of Serbia, 21000 Novi Sad, Serbia

**Keywords:** *Schizophyllum commune*, fungal polysaccharides, seed biopriming, *Pisum sativum* L., antioxidant enzymes, plant biostimulants, PCA

## Abstract

This study investigated the biostimulant potential of endo- and exo-polysaccharides (PSHs) obtained from two *Schizophyllum commune* strains originating from Italy (ITA) and Serbia (SRB), with a primary focus on their ability to modulate the antioxidant defense and biochemical responses of *Pisum sativum* L. seedlings under optimal and drought conditions. The PSH fractions were structurally characterized by complementary spectroscopic and microscopic approaches, revealing strain- and drying-dependent differences in their structural features. The characterized PSHs were subsequently applied as seed biopriming agents, and pea seedlings were grown under two conditions: optimal growth and drought stress. Their effects were evaluated by analyzing enzymatic and non-enzymatic antioxidant responses, together with selected physiological and biochemical traits. Among the tested treatments, exo-PSHs differentially modulated antioxidant enzymes, with SRB primarily enhancing peroxidase and glutathione peroxidase and ITA increasing ascorbate peroxidase and catalase activity. SRB PSHs, particularly exo-PSH, showed the most pronounced effects on antioxidant-related responses, enhancing ABTS scavenging activity and altering DPPH capacity by approximately 35% compared with the control. In addition, endo-PSH from the ITA strain increased chlorophyll content by 26.12% and proline levels by 1.5-fold, indicating a distinct strain-dependent effect on plant physiological responses, particularly under stress conditions. LC–MS/MS profiling of plant MeOH extracts revealed a diverse range of secondary metabolites, including phenolic compounds, e.g., liquiritigenin, ferulic, and protocatechuic acids, which may contribute to the observed antioxidant responses. Multivariate analysis further supported clear biochemical differentiation among PSH treatments and growth conditions, confirming that the responses were dependent on both PSH origin and environmental conditions. Overall, these findings demonstrate that structurally distinct *S. commune* PSHs can differentially modulate antioxidant defense and key biochemical traits in pea seedlings, supporting their potential as sustainable fungus-derived biostimulants for enhancing plant resilience under both optimal and drought conditions.

## 1. Introduction

In the context of sustainable food production systems, improving plant resilience to environmental stress while reducing dependence on synthetic agrochemicals has become an important research priority [1,2]. Drought is among the major constraints limiting crop establishment and productivity, particularly during early plant development, and has therefore increased interest in sustainable strategies capable of improving plant stress tolerance. Biopriming, which involves treating seeds with beneficial microbes or their metabolites, is one such approach that can precondition seeds and seedlings for improved responses to subsequent environmental stress [3,4]. During biopriming, biochemical and molecular processes associated with stress perception, redox homeostasis, and defense responses can be modulated, potentially resulting in enhanced antioxidant capacity and greater stress tolerance during subsequent plant development [4,5]. In particular, the activation of antioxidant enzymes and stimulation of phenolic and other protective metabolites may contribute to limiting oxidative damage caused by drought-induced reactive oxygen species [4,5].

Pea (*Pisum sativum* L.) represents a highly significant crop group and a relevant experimental system for studying early plant responses to drought stress. Its productivity and seedling development can be strongly affected by water limitation, which induces oxidative stress and triggers coordinated physiological and biochemical defense mechanisms [6]. Moreover, because legumes contribute substantially to plant-based protein production and sustainable agricultural systems, improving their resilience to drought is of particular agronomic relevance. Previous studies have shown that polysaccharides (PSHs) of microbial origin can act as effective seed treatments and promote seedling growth and stress-related responses of *Triticum vulgare* and *Phaseolus vulgaris* [7].

Fungi are a particularly interesting source of PSHs because they produce structurally diverse bioactive polymers, including β-glucans and heteropolysaccharides, which have been associated with antioxidant and antimicrobial properties, among others [8,9,10,11]. Compared with conventional chemical elicitors, fungus-derived PSHs may therefore represent a promising biobased resource for the development of sustainable plant biostimulants.

The cosmopolitan species *Schizophyllum commune* Fr. 1815 (fam. Schizophyllaceae, ph. Basidiomycota), commonly recognized as the white-rot fungus, stands out for its ability to synthesize a wide range of bioactive compounds, including lignocellulolytic enzymes, peptides, PSHs (e.g., schizophyllan (SPG), glucomannan), and bioethanol [12,13,14,15,16,17,18]. Among fungal PSHs, those produced by *S. commune*, particularly the β-glucan SPG, are notable for their unique triple-helix structure and high biological activity, and have attracted considerable interest in biotechnology, food, agriculture, and functional biomaterials [19,20]. Moreover, submerged cultures of filamentous fungi offer a unique and controlled environment for the targeted production of diverse metabolites, enabling systematic analysis of secreted bioactive compounds with broad industrial relevance [21,22,23,24]. The cultivation process allows for partial manipulation of metabolite production by altering environmental factors such as substrate composition, pH, temperature, and aeration throughout the growth period [22,25].

While various biostimulants and biofertilizers have gained traction in recent years, the potential of fungal PSHs as seed biopriming agents remains insufficiently explored. In our previous study, we demonstrated for the first time that exo- and endo-PSHs obtained from submerged cultures of two *S. commune* strains of different geographic origin (Italy, ITA, and Serbia, SRB) positively affected seed germination and early seedling development of *P. sativum* L. under both optimal and drought conditions [14]. The magnitude of these effects varied according to PSH type, strain origin, and growth conditions, suggesting that fungal PSHs may differentially modulate plant responses rather than act through a uniform biostimulatory mechanism. These findings provided the first evidence of the biopriming potential of *S. commune* PSHs in pea and raised the question of whether the observed effects on germination and seedling growth are associated with changes in antioxidant defense, osmotic adjustment, membrane stability, and plant secondary metabolism. However, the influence of PSH biopriming on the enzymatic and non-enzymatic antioxidant systems, chlorophyll and proline accumulation, lipid peroxidation, and the phytochemical composition of pea seedlings has not yet been elucidated.

Therefore, the primary aim of the present study was to determine whether exo- and endo-PSHs obtained from two geographically distinct *S. commune* strains can modulate antioxidant defense and stress-related physiological responses in *P. sativum* seedlings under optimal and drought conditions. Hence, we evaluated antioxidant enzyme activities and non-enzymatic antioxidant capacity, together with chlorophyll, proline, and lipid peroxidation as indicators of physiological status and oxidative stress, and characterized changes in the phenolic profile of seedling extracts. The structural characteristics of the fungal PSHs used for biopriming and multivariate relationships among the measured responses were also examined to explore potential factors underlying the differential biostimulatory effects.

## 2. Materials and Methods

### 2.1. Fungal Material

Two dikaryotic strains of the wild *S. commune* were gathered, one near Bologna (Italy, ITA) in 2016, and the other in Zmajevac (Fruška Gora Mountain, Serbia, SRB) in 2012. Mycelia (M) from both strains of *S. commune* were isolated, cultivated, preserved, and deposited in the FUNGICULT collection (ProFungi laboratory, Department of Biology and Ecology, Faculty of Sciences, University of Novi Sad, Serbia), with reference numbers 0043 for the SRB strain and 0047 for the ITA strain, as published in Mišković et al. [14,26].

### 2.2. Molecular Identification of Species—ITS Sequencing

For morphological identification, the DNA sequence was determined for each fungal isolate examined. Fungal material was pre-pulverized with liquid nitrogen (~100 mg) and collected for total genomic DNA extraction using a ZR fungal/bacterial DNA MiniPrepTM kit (Irvine, USA) according to the manufacturer’s protocol [27]. One ITS II gene region (internal transcribed spacer 1, partial sequence; 5.8S ribosomal RNA gene and internal transcribed spacer 2, complete sequence; and large subunit ribosomal RNA gene) was amplified [28]. The primers used to amplify the above regions and their corresponding PCR profiles were the ITS1-F TCCGTAGGTGAACCTGCGGITS1 and ITS2 regions, separated by the 5.8S genome.ITS4-RTCCTCCGCTTATTGATATGC

PCR was performed at a final volume of 25 μL, as described by Six et al. [28]. Each PCR reaction mixture (25 μL total volume) consisted of 10.5 μL of ultrapure water without DNase/RNase (Gibco, UK), 12.5 μL of Fast Gene Taq (NIPPON Genetics, Europe) ready mix with a dye of 250 × 50 μL, 0.5 μL of each primer (10 μM), and 1 μL of DNA extract. The PCR conditions were as follows: an initial denaturation cycle at 95 °C for 4 min, followed by 35 cycles of denaturation at 95 °C for 30 sec, annealing at 52 °C for 60 sec, extension at 72 °C for 60 sec, and a final extension cycle at 72 °C for 10 min. PCR products were purified using an EXTRACTME DNA clean-up kit according to the manufacturer’s protocol (BLIRT, Poland). Purified PCR products were sequenced as a commercial service at Eurofins Scientific (Germany). Homologies for the obtained sequences were searched against the GenBank database using the BLASTN program of the National Center for Biotechnology Information (NCBI), and all chromatograms were manually checked for nucleotide positions. Ordered and reference sequences of strains from the GenBank database were aligned using Clustal W, implemented in BioEdit 7.2.5 software.

### 2.3. Isolation of Fungal PSHs

The PSH extracts were prepared following the procedure outlined by Chen et al. [29], with specific adjustments as per Mišković et al. [14,26]. Both *S. commune* strains (SRB and ITA) were cultivated on malt agar at 26 °C for 12 days, then transferred to liquid medium and grown under submerged conditions (120 rpm, 26 °C) for 14 days. The medium consisted of peptone, glucose, maltose, fructose, xylose, yeast extract, dipotassium hydrogen phosphate (K_2_HPO_4_), magnesium sulfate heptahydrate (MgSO_4_ × 7H_2_O), vitamin B1, and distilled water (dH_2_O). After filtration, exo-PSH was obtained from the filtrate by ethanol precipitation, centrifugation, drying, and rehydration in dH_2_O. The mycelial biomass was frozen, lyophilized, ground, and repeatedly hydrated and dried before centrifugation. The resulting supernatant contained the endo-PSH.

### 2.4. Characterization of Fungal PSHs

#### 2.4.1. Congo Red Assay

The interaction of Congo red with PSHs was used to evaluate the conformational characteristics of exo-PSH and endo-PSH using a combined and slightly optimized protocol derived from previously published methods [30,31], adapted in this study for the specific properties of the investigated polysaccharide samples. PSH solution (2 mL, 2 mg/mL) was mixed with an equal volume (2 mL) of 91 μmol/L Congo red reagent (Sigma-Aldrich, Merck Group, Germany). The next step involved adding 1 mL of NaOH solution with a concentration range of 0 to 0.7 mg/mL. Congo red reagent (Sigma-Aldrich, Merck Group, Germany) was used as a control. Absorbance was measured using a spectrophotometer (Multiskan GO Thermo Scientific, Finland) at the following wavelengths: 400, 440, 480, 520, 560, and 600 nm. A curve depicting the dependence of the wavelength of maximum absorption and NaOH concentration was generated.

#### 2.4.2. Scanning Electron Microscopy (SEM)

Morphological analysis was performed using a JEOL JSM 6460 LV scanning electron microscope (SEM) at the University Center for Electron Microscopy in Novi Sad, Serbia. Samples of exo-PSH from the ITA and SRB strains were dried by two different methods, air-drying and lyophilization, then directly applied onto double-sided adhesive carbon tape substrates. Subsequently, a 90 s gold coating was applied using a BAL-TEC SCD 005 sputter coater at a distance of 50 mm from the plasma source at 30 mA. Photographs were taken at an accelerating voltage of 20 kV under high-vacuum conditions.

#### 2.4.3. NMR Analysis

NMR spectra were recorded on a Varian/Agilent 400 MHz spectrometer using a 5 mm broad-range probe. Spectra were obtained at 25 °C in DMSO-d6 (15 mg/mL) with sodium-2,2-dimethyl-2-silapentane-5-sulfonate (DSS) as an internal reference standard. Due to the low solubility of the tested samples in dimethyl sulfoxide (DMSO), certain samples underwent enzymatic treatment prior to NMR analysis. The enzymatic treatment involved the use of DNase, RNase, and protease, followed by dialysis and lyophilization of the samples [32].

#### 2.4.4. Raman Spectroscopy

Raman spectra of the samples were recorded on a DXR Raman microscope (Thermo Scientific, USA) equipped with an optical microscope and a CCD detector. The spectra were recorded using a high-brightness frequency-stabilized single mode diode laser with an excitation wavelength (λ_exc_) of 780 nm. The spectra were recorded using laser power of 24 mW on the sample, 10 s of exposure time and 20 exposures per spectrum, a grating with 400 lines/mm, a 50 μm slit spectrograph aperture, and 0.5 min photobleaching time. The laser beam was focused on the sample placed on the glass slide, using an objective with ×10 magnification and an X–Y motorized sample stage. The spectra were corrected for fluorescence using the OMNIC software package. The spectra were recorded immediately after taking the samples from the closed vessel stored in the refrigerator.

#### 2.4.5. ATR-FTIR Spectroscopy

FTIR spectra of the samples were recorded in the mid-infrared region (4000–525 cm^−1^) using a Nicolet iS 20 FTIR spectrometer (Thermo Fisher Scientific, Waltham, MA, USA) operated in ATR mode. The instrument was equipped with a monolithic diamond crystal, and measurements were performed at a resolution of 4 cm^−1^ with 16 scans.

#### 2.4.6. TGA–DTA Analysis

Thermogravimetric and differential thermal analyses (TGA–DTA) of the investigated samples were obtained using an SDT Q600 device (TA Instruments, USA). The program was set to a 10 °C min^−1^ rate, from 20 to 800 °C in N_2_, with a flow rate of 80 mL min^−1^.

### 2.5. Seed Priming

Pea seeds were processed according to the methodology outlined in Mišković et al. [14]. Garden pea (*Pisum sativum* L.) cv. Dunav, developed at the Institute of Field and Vegetable Crops, Novi Sad (Serbia), was used. Seeds were produced at Rimski Šančevi in 2022. Seeds were surface-sterilized with 5% NaClO and rinsed three times with dH_2_O. Priming was performed by soaking seeds for 6 h in dH_2_O (hydropriming) or 1% (10 mg/mL) PSH solutions (exo-PSH or endo-PSH) from both fungal strains in a 1:5 (*w*/*v*) ratio [33,34]. Control seeds remained unprimed. The experimental design comprised six seed-treatment groups: (i) unprimed control, (ii) hydropriming (HP) (dH_2_O), (iii) ITA exo-PSH, (iv) ITA endo-PSH, (v) SRB exo-PSH, and (vi) SRB endo-PSH. Each seed-treatment group was subsequently evaluated under both optimal and drought conditions, resulting in 12 experimental groups in total (Table 1). After treatment, seeds were rinsed and air-dried to their initial weight.

### 2.6. Plant Growth Under Optimal and Drought Conditions

Following the biopriming treatment, pea seeds were sown in sterilized sand as a growth substrate. Five plants were grown per pot, with three pots used for each treatment. The pots were randomly arranged within a controlled-environment growth chamber to minimize positional effects. Plants were grown for 21 days in a controlled-environment chamber (JSR, Republic of Korea) at 20 °C under a light intensity of 15,000 lux and a 16 h light–8 h dark photoperiod.

Two growth conditions were established: optimal condition and drought stress. Under optimal conditions, the sterilized sand was moistened with dH_2_O and maintained at an adequate soil moisture content throughout the 21-day growth period. Drought stress was simulated by supplying the substrate with a 20% (*w*/*v*) polyethylene glycol (PEG) 6000 solution (Sigma-Aldrich, St. Louis, MO, USA). The corresponding solution (1125 mL) was added to 20 L of sterilized sand to adjust the substrate moisture content and maintain the desired water content of 5.63%. The PEG 6000 solution was continuously used for irrigation throughout the experimental period to maintain the imposed drought conditions.

At the end of the 21-day growth period, plant material was harvested for subsequent physiological, biochemical, and antioxidant analyses. Fresh plant material was immediately processed for the determination of chlorophyll, enzyme activities, and proline content. For analysis requiring dried material, plant samples were collected and dried. The dried samples were subsequently stored in paper bags in the dark at room temperature until further analysis.

### 2.7. Preparation of Plant Extracts

After biopriming, 0.50 g of fresh leaf mass of *P. sativum* L. was weighed on an analytical balance and macerated with the help of liquid nitrogen and 3 mL of extraction phosphate buffer (pH 7.5). Afterward, the extracts were centrifuged (Centrifuge 5424 R, Eppendorf, Germany) (13,000 rpm, 20 min), and the supernatant was used for further analyses. Separately, methanolic (MeOH) extracts of peas were prepared from the dried mass of the aerial parts and the dried mass of the roots. Extraction of 1 g of aerial parts and 1 g of plant roots in 80% MeOH took 24 h on a shaker (IKA, Germany) at room temperature. Afterward, the extracts were centrifuged (10,000 *g*, 4 °C, 20 min; Centrifuge 5424 R, Eppendorf, Germany) and the supernatant was used as an extract for further analyses. The plant extracts were stored at +4 °C before analysis, with a final concentration of 20 mg/mL.

### 2.8. Chemical Characterization of Pea Extracts

#### 2.8.1. Total Chlorophyll and Carotenoid Content

The total chlorophyll and carotenoid content in lyophilized plant samples was determined according to the method of Kamble et al. [35]. A fresh leaf mass (0.1 g) was extracted in a chilled mortar with the addition of quartz sand, 80% acetone, and MgCO_3_. After filtration, the absorbance of the leaf extracts was measured at wavelengths of 662 nm, 644 nm, and 440 nm, using 80% acetone as a blank.

The pigment content in the leaves was calculated using the following formulas:

Chlorophyll a (Ca) = 9.784 × A_662_ − 0.990 × A_644_ (mg/mL)

Chlorophyll b (Cb) = 21.426 × A_644_ − 4.65 × A_662_ (mg/mL)

Chlorophyll a + b (Ca + Cb) = 5.134 × A_662_ + 20.436 × A_644_ (mg/mL)

Carotenoids (C) = 6.695 × A_440_ − 0.268 × (chlorophyll a + b) (mg/mL)

The concentration of pigments was expressed in mg/g of dried leaf mass using the formula:

C = C_1_ × V × r/m

where *C*_1_ is the mass concentration of the pigment (mg/L), *V* is the volume of the filtrate (L), *r* is the dilution factor, and *m* is the sample mass (g).

#### 2.8.2. Total Protein, Phenolic, Flavonoid, and Proline Content (TPR, TPC, TFC, PRO)

TPR was determined in plant extracts obtained from fresh pea mass by the colorimetric dye-binding method [36]. The protein concentration was read from the calibration curve and determined for each plant extract in three replicates, and then the mean value (mg eq. BSA/g d.w.) was calculated. TPC was determined in the extracts of the aerial part and roots of peas obtained from the dry mass (concentration range from 20 to 1.25 mg/mL) as per Singleton et al. [37] using the method described by Mišković et al. [26]. TFC in plant extracts obtained from dry mass was determined using the spectrophotometric method described by Chang et al. [38]. The results are presented as the mean value of three measurements ± SD (mg eq. quercetin/g d.w.). PRO was determined from fresh aerial parts of the plant [39]. Absorbance was read at a wavelength of 520 nm, L-proline (Pro) was used as a standard compound, and results are expressed as mg eq. Pro/g d.w.

#### 2.8.3. LC–MS/MS Profiling of Selected Compounds

Quantification of selected compounds in MeOH extracts prepared from the dry mass of pea aerial parts and roots was performed using an LC–MS/MS approach [40]. Reference standards of investigated compounds were obtained from Fluka Chemie GmbH (Buchs, Switzerland), Chromadex (Santa Ana, CA, USA), Roth Chemie GmbH (Karlsruhe, Germany), or Sigma-Aldrich (Steinheim, Germany). Compound stock solutions were prepared in DMSO, and two standard mixes were prepared by mixing them. Calibration curves were in the range 1.53–12,500 ng/mL (standard mix compositions are given in Supplementary Materials Tables S1 and S2). Analysis was performed using an Agilent Technologies 1200 series high-performance liquid chromatograph coupled with an Agilent Technologies 6410A Triple Quad tandem mass spectrometer with an electrospray ion source and controlled by Agilent Technologies MassHunter Workstation software—Data Acquisition (ver. B.03.01) (Agilent Technologies, Santa Clara, CA, USA). The sample or standard (5 µL) was injected, and components were separated on a Zorbax Eclipse XDB-C18 rapid resolution column (50 mm × 4.6 mm, 1.8 µm) heated to 50 °C. The mobile phase consisted of 0.01% HCOOH in water (A) and MeOH (B), with an applied flow of 1 mL/min in gradient mode (0 min 30% B, 6 min 70% B, 9 min 100% B, 12 min 100% B, re-equilibration time 3 min; total analysis time 15 min). Eluted components were detected by MS using the following ion source parameters: nebulization gas (N_2_) pressure 40 psi, drying gas (N_2_) flow 9 L/min and temperature 350 °C, capillary voltage 4 kV, negative polarity. Data were acquired in dynamic MRM mode using the optimized compound-specific parameters (retention time, precursor ion, product ion, fragmentor voltage, collision voltage; Supplementary Materials Tables S1 and S2). For all compounds, peak areas were determined using Agilent MassHunter—Qualitative Analysis (ver. B.06.00). Calibration curves were plotted and sample concentrations calculated using OriginLabs OriginPro (ver. 2019b).

### 2.9. In Vitro Antioxidant Activity of Pea Extracts

#### 2.9.1. Determination of Enzymatic Activity

For the determination of enzyme activity, fresh extracts obtained from the aerial parts of pea plants were used, while for the final recalculation of the activity of the tested enzymes, the protein concentration determined in the extracts using the Bradford [36] method was used. The activity of pyrogallol peroxidase (POD) was determined by a spectrophotometric method based on the catalytic role of this enzyme in the redox reaction, during which the oxidation of pyrogallol, which serves as a substrate, and the reduction of H_2_O_2_ occur [41]. The activity of guaiacol peroxidase (GPX) was determined by the method of Chance and Maehly [42], based on the reduction of H_2_O_2_ using guaiacol as an electron donor, which in the presence of enzymes and H_2_O_2_ turns into yellow tetraguaiacol. The reaction mixture contained the plant extract, 50 mM potassium phosphate buffer (pH 7), 5 mM H_2_O_2_, and 18 mM guaiacol such that the total volume was 1 mL. Ascorbate peroxidase (APX) activity in plant extracts was determined spectrophotometrically by measuring absorbance at a wavelength of 290 nm every 10 sec for 3 min [41]. Catalase (CAT) activity was determined spectrophotometrically by monitoring the drop in absorbance at a wavelength of 240 nm every 10 sec for two min according to Aebi [43].

#### 2.9.2. Determination of Non-Enzymatic Activity

Neutralization of DPPH activity of plant extract derived from dry mass was determined according to the method of Espín et al. [44]. The absorbance intensity was read spectrophotometrically at 515 nm after incubation in the dark for 30 min. The results are expressed as the mean of three determined IC_50_ values ± standard deviation (SD) (μg/mL). The ABTS radical-scavenging capacity of plant extracts derived from dry mass was determined according to the method conducted by Arnao et al. [45]. The capacity of aerial pea parts (from dry mass) to “capture” the hydroxyl radicals was examined according to Halliwell et al. [46], and the results are expressed as the mean of three determined IC_25_ values ± SD (μg/mL). Inhibition of lipid peroxidation (LP) of plant extracts of aerial parts (from dry mass) was tested using the TBA method [47], and the results are expressed as the mean value of three determined IC_25_ values (μg/mL) ± SD.

IC_50_ and IC_25_ values were determined using concentration–response curves generated in OriginPro. The percentage of radical neutralization or inhibition was plotted against the corresponding extract concentration, and the data were fitted using nonlinear regression. For assays in which 50% inhibition or radical neutralization was not reached within the tested concentration range, the results are expressed as IC_25_, defined as the concentration of extract required to achieve 25% inhibition or radical neutralization. IC_25_ was used as an alternative indicator when a reliable IC_50_ value could not be determined. Since IC_25_ is less commonly reported than IC_50_ in the literature, this difference should be taken into consideration when comparing the present results with previously published data.

### 2.10. Statistical Analysis

The results are expressed as means of three technical replicates ± SD. All data had normal distribution and were subjected to appropriate univariate analysis. Two-way analysis of variance (ANOVA) was performed to evaluate the effects of growth condition (optimal vs. drought), PSH treatment/priming type, and their interaction on each measured parameter, including TPC, TFC, TPR, PRO, in vitro enzymatic activities, and antioxidant parameters (OH, DPPH, ABTS, FRAP, and lipid peroxidation). Thus, each parameter was analyzed separately using the same two-factor ANOVA model. Significant differences identified by ANOVA were further analyzed using Tukey’s HSD post hoc at a significance level of 95% (*p* < 0.05). Correlation analysis was performed using Pearson’s product-moment correlation. Statistical analysis was performed in IBM SPSS statistical software (version 22.0 for Windows) and Statistica software (version 12.01). PCA analysis (principal component analysis) was also performed using Past 4 Project software (version 1.0.0.0). OriginPro 8 was used to prepare selected graphic representations.

## 3. Results and Discussion

### 3.1. Phylogenetic Analysis

Molecular identification of the fungal strains was performed by amplifying the complete sequence of the internal transcribed spacer ITS 1, 5.8 S r RNA genes, and the ITS 2. The sequence of the amplified DNA fragment was identified as follows. Strains were marked 0043 and 0047, and sequences of the amplified DNA fragment were identified as *S. commune* species (100%) according to the nucleotide database of the National Center for Biotechnology Information (NCBI), as presented in Figure S1. While ITS sequencing confirms species identity, further analysis of the mating-type loci that govern sexual compatibility in this tetrapolar basidiomycete could provide deeper insight into the strain-specific physiological differences observed in this study. Tools such as the recently developed mating-type imputation (MTI) method offer an efficient approach for such genetic characterization [48].

The phylogenetic tree (Figure S2) was constructed using the neighbor-joining method, and the distances were calculated using the Kimura two-parameter model. The percentage of replicate trees in which related taxa clustered in the initial test (1000 replicates) is shown next to the branches. The tree is drawn to scale, with branch lengths in the same units as the evolutionary distances used to infer the phylogenetic tree. The analysis included four nucleotide sequences. All positions with less than 95% site coverage were eliminated. That is, less than 5% of alignment gaps, missing data, and ambiguous bases were allowed at any position.

### 3.2. Characterization of Exo-PSHs and Endo-PSHs

#### 3.2.1. Congo Red Test

Given that the conformation of PSHs can significantly influence their bioactivity [29], the interaction of Congo red with exo- and endo-PSHs from both ITA and SRB strains was evaluated within a wavelength range of 400 to 600 nm (Figure S3). PSHs possessing a triple-helix conformation can form a complex with Congo red, leading to a bathochromic shift (i.e., an increase in the maximum absorbance wavelength, λmax) compared to the control (Congo red alone). However, this shift can be affected by environmental factors such as pH or alkaline conditions as baseline conditions.

The results showed that λmax varied between 400 and 520 nm, depending on the type of PSHs analyzed and the concentration of NaOH. For endo-PSH from the ITA strain, λmax increased to 485 nm at a NaOH concentration of 0.4 mg/mL, followed by a decrease and flattening of the curve (λmax = 480 nm) despite further increases in NaOH concentration. This suggests that the triple-helix conformation was disrupted under strongly basic conditions, indicating total loss of the triple-helix conformation of this PSH isolated from mycelial biomass. A similar trend was observed for exo-PSH isolated from extracellular filtrate from the same strain, indicating that both exo- and endo-PSHs in the ITA strain possess a triple-helix structure. Previous studies have reported that SPG, the predominant β-glucan produced by *S. commune*, maintains its triple-helix conformation in aqueous and mild alkaline conditions, but transitions to a random coil structure at higher NaOH concentrations [49,50]. Therefore, it can be inferred that the exo- and endo-PSHs isolated from the ITA strain are β-glucans, most likely SPG, exhibiting characteristic triple-helix conformations under mild alkaline conditions.

Conversely, an opposite pattern was observed for the PSHs derived from the SRB strain. Specifically, no significant changes in λmax were detected during Congo red complexation, indicating that both exo- and endo-PSHs from this strain likely lack a triple-helix conformation. This observation aligns with the literature and prior investigations that have demonstrated that hetero-PSHs are generally unable to form stable triple helices [29,51]. Accordingly, the analyzed SRB-derived exo- and endo-PSHs are likely hetero-PSHs that adopt a random coil or linear conformation.

Further support for this interpretation is the elemental microanalysis, which detected trace amounts of proteins and aromatic compounds in the isolated exo- and endo-PSHs [14]. These findings suggest that the SRB-derived PSHs may form complexes with other biomolecules, potentially influencing their structure and preventing the formation of ordered helicoidal arrangements. Also, conformational changes may indicate modifications in molecules’ organization that can influence PSH stability and their ability to interact with biological systems, thereby potentially affecting their observed bioactivity [52].

#### 3.2.2. SEM Analysis

**Figure 1:**
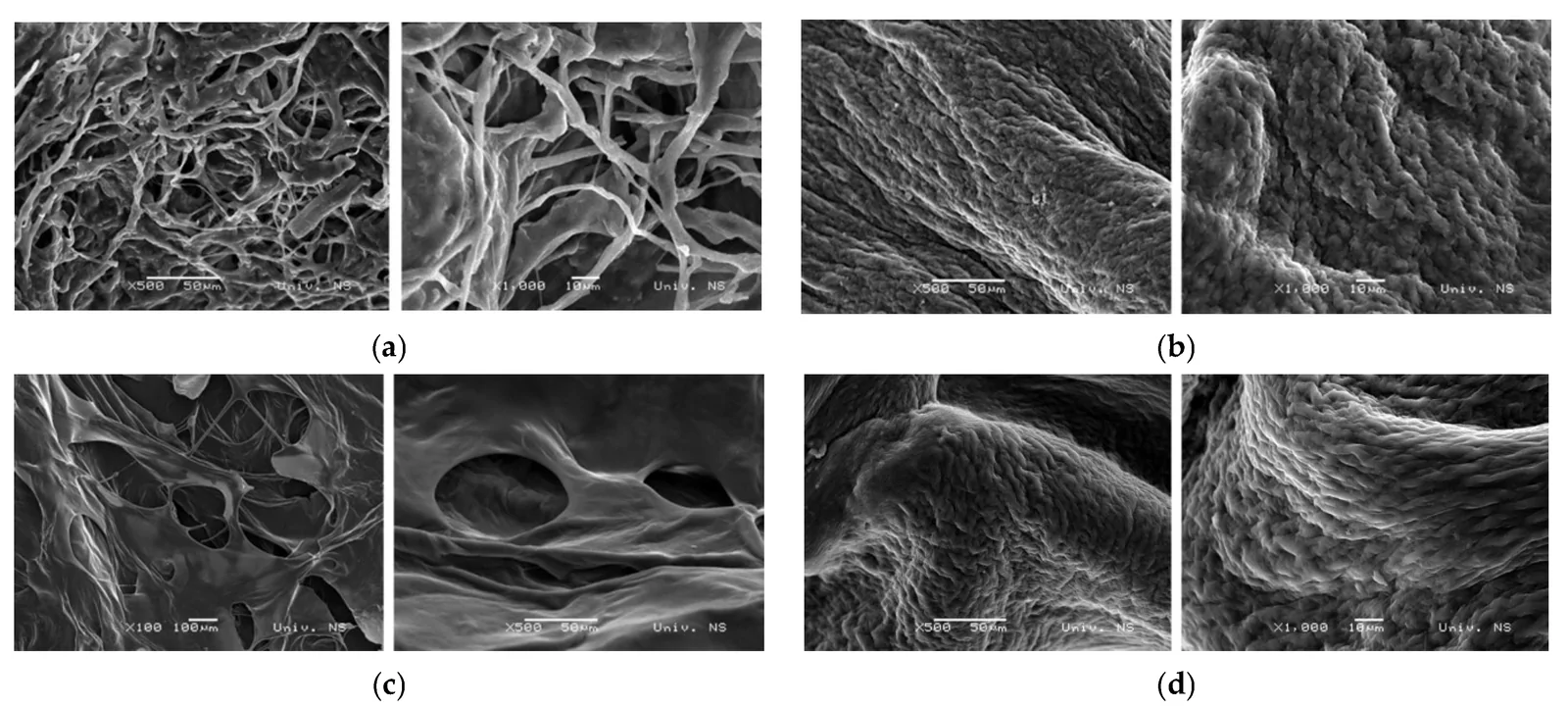
SEM images of exo-PSH from *S. commune* strains: (**a**) ITA lyophilized sample (500× and 1000× magnification), (**b**) ITA air-dried sample (500× and 1000× magnification), (**c**) SRB lyophilized sample (100× and 500× magnification), (**d**) SRB air-dried sample (500× and 1000× magnification).

Exo-PSHs isolated from both strains of *S. commune* were observed using a scanning electron microscope in order to analyze the topography of these polymers (Figure 1).

One part of the samples was air-dried, while the other was lyophilized and freeze-dried to assess the effect of the drying method on the resulting scanning micrographs, as shown in Figure 1a–d.

A variety of microstructural features were observed in exo-PSH samples, particularly in relation to the drying method used. Surface morphologies differed markedly in shape and texture, with air-dried samples (Figure 1b,d) exhibiting significantly rougher surfaces compared to those that were lyophilized. The air-dried samples depicted in Figure 1b,d exhibited notably rougher surfaces in comparison to the lyophilized PSHs. This variation is likely due to the denaturation of proteins and the disruption of hydrogen bonding and hydrophobic interactions during air-drying [53]. In addition to the drying method, structural differences were also noted between exo-PSH samples derived from different fungal strains. Specifically, the air-dried exo-PSH from the SRB strain exhibited a larger and more compact structure, while the ITA strain presented with finer and more delicate microstructures, which can be assigned to differences in strain genetic background.

Specifically, the air-dried sample from the SRB strain displayed a larger and more compact structure, while the ITA strain presented with finer and more delicate microstructures. These observations are consistent with prior research, which has shown that both the origin of a PSH and the applied processing methods can significantly affect the morphology and structural integrity of fungal PSHs [29,54,55].

On the other hand, when it comes to lyophilized samples, a smooth surface with a mesh structure containing irregular ruptures is clearly visible in both strains (Figure 1). SEM of exo-PSH, isolated from *S. commune* originating from China, also showed a homogeneous structure of this polymer with a smooth and shiny surface [56]. Additionally, exo-PSH originating from *S. radiatum* had a smooth and thin surface, with pores present [23]. The smooth surface is most likely a consequence of the mutual connection between the hydrogen proton donor –OH group of the exo-PSH and the hydrogen acceptor –NH group of the protein [53], but also stronger intermolecular interactions due to the high molecular mass [23], which were preserved during the lyophilization procedure. The network structure of exo-PSH was created due to hydrogen interactions between individual PSH molecules, while the different degree of porosity is due to hydrogen bonds between C–O fragments of PSHs and N–H fragments of proteins present [53,57]. The higher porosity observed in the SRB strain (Figure 1) and Congo red test where the helicoidal structure was negated together confirm that the analyzed exo-PSH isolated from the SRB strain is probably a PSH complex (most likely hetero-PSHs), proteins and secondary metabolites. Also, it can be concluded that in addition to the different origins of exo-PSH, the method of drying, i.e., preparing the samples, has an obvious influence on the PSH microstructure. Such morphological differences may affect the exposure of functional groups and the hydration properties of PSHs, thereby influencing their interaction with plant surfaces and potentially contributing to the observed variation in biological activity.

#### 3.2.3. NMR Analysis of Polysaccharides

^1^H NMR spectra were recorded for exo-PSHs and endo-PSHs from both *S. commune* strains, as shown in Figure S4. Chemical shifts are expressed in ppm.

The spectra were quite similar, although the signals were not clearly separated, which was attributed to insufficient solubility of the samples in DMSO. However, signals in the 3–5.5 ppm region corresponding to PSHs can be clearly observed. The peak at 2.5 ppm originates from the solvent DMSO, while the peak from water present in the sample was located at 3.4 ppm and overlapped with the broad peak for sugar protons. Multiple peaks in the 1–3 ppm region were observed, corresponding to a trace sugar-free aliphatic compound, consistent with a previously published NMR spectrum of PSHs isolated from *S. commune* originating from Avala (Serbia) [58]. Additionally, the signals in that region most likely originate from the present proteins [59]. Peaks in the 6.5–9 ppm region confirm the presence of aromatics in the sample, which was proven by previous analyses (Congo red, SEM) and available literature data [14,58]. Moreover, the intensity of these peaks is significantly lower compared to the peaks in the 3–5.5 ppm region, which is consistent with the dominant PSH structure of the extract.

Given the complex composition of the sample, assigning the peaks in the ^13^C NMR (Figure S5) spectrum is challenging. However, the most intense peaks were observed in regions typical of PSHs, specifically 60–80 ppm, which could be attributed to C2, C3, C4, C5, and C6. In addition, in the regions of 20–40 ppm and 130 ppm, low-intensity signals were present that could be attributed to proteins and aromatics, respectively [60,61].

**Figure 2:**
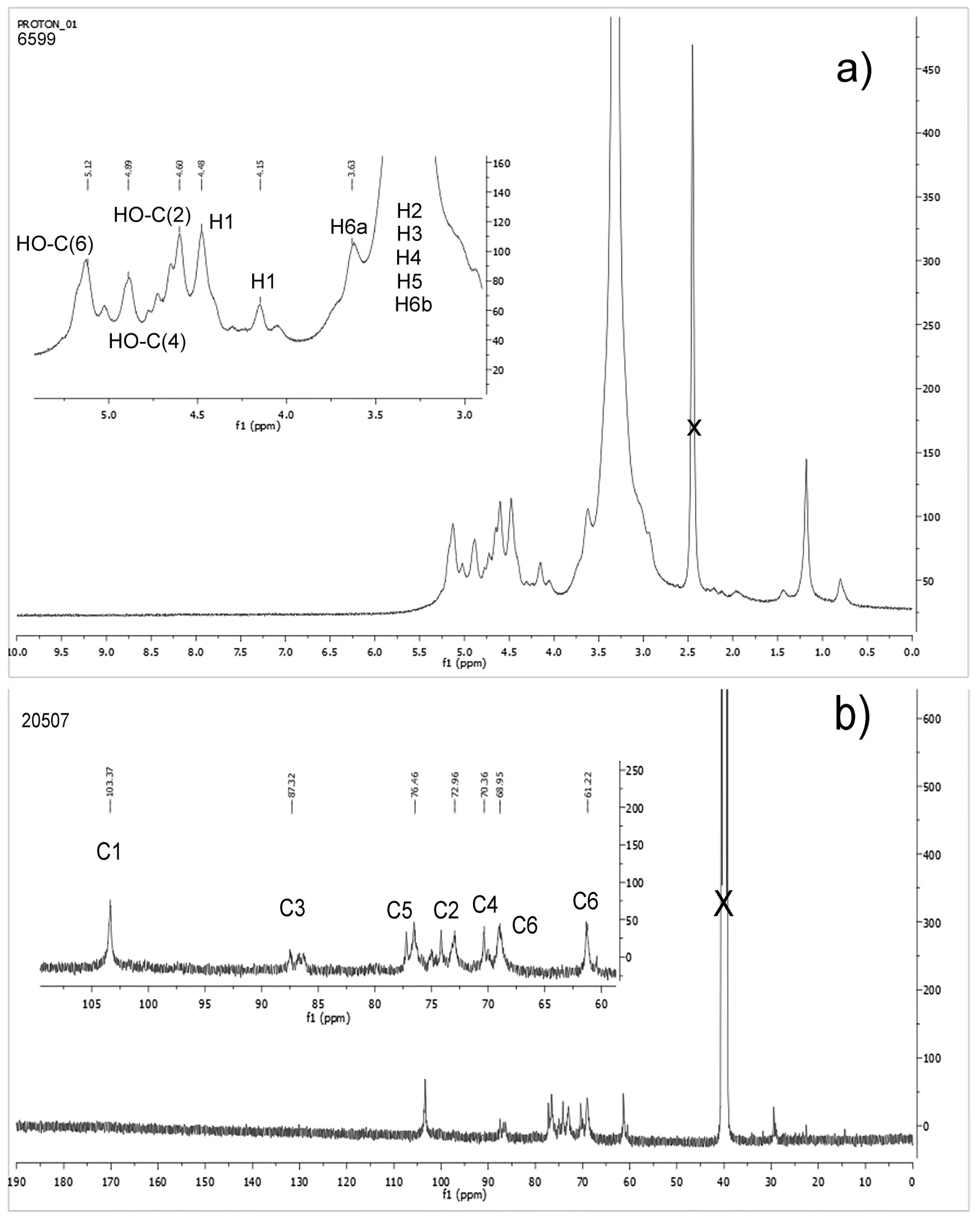
^1^H NMR (**a**) and ^13^C NMR (**b**) spectra of endo-PSH SRB in DMSO.

To obtain a clearer picture of the structural characteristics of PSHs, the samples were subjected to enzymatic treatment to remove non-carbohydrate co-extracted components. In ^1^H NMR and ^13^C spectra of enzymatically treated samples, individual peaks were better separated and the absence of signal in the aromatic region was observed (Figure 2 and Figure S6).

The NMR spectra of endo-PSH SRB extract (Figure 2) were compared with the literature data for the SPG from *S. commune*, which is structurally a series of D-glucose units with a β-1,3 bond in the backbone and branching in the β-1,6 position [62,63]. In the ^1^H NMR spectrum (Figure 2a), a broad peak observed in the region 3.0–3.5 ppm corresponds to protons H2 to H6. According to the literature data, the peak at 4.5 ppm is attributed to the H1 proton from the main sequence, and the peak at 4.2 ppm to the H1 proton from the branching [63]. The intensity ratio of these two peaks obtained by integration is 1:0:4, which is approximately in agreement with literature data that state 0.33 as the degree of branching. Also, the isolated peak at 3.6 ppm was assigned to the H6a proton. The peaks at 4.65, 4.94 and 5.18 ppm most likely correspond to the protons of the hydroxyl groups at positions 2,4 and 6.

In the ^13^C NMR spectrum of the endo-PSH SRB extract (Figure 2b), a signal at 103.37 ppm in the anomeric carbon region indicates the presence of a β-glycosidic bond. Peaks at 61.22 and 68.95 ppm were assigned to free C6 and bound C6 from branching, respectively. The positions of the remaining peaks (C2—72.96 ppm, C3—87.32 ppm, C4—70.36 ppm, C5—76.46 ppm) are consistent with literature data for β-glucan [63].

#### 3.2.4. Raman Spectroscopy of Polysaccharides

**Figure 3:**
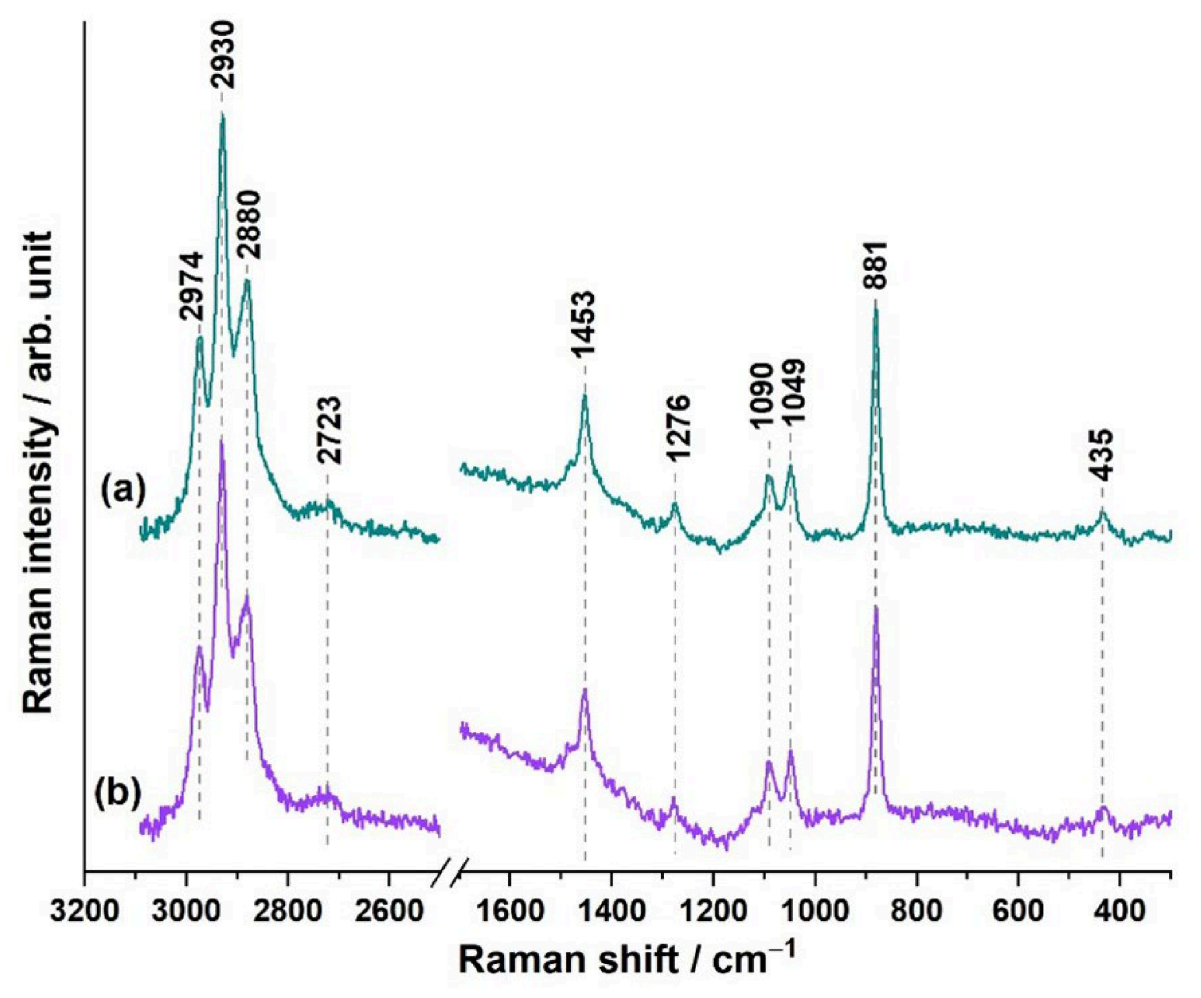
Raman spectra of the samples: (a) exo-PSH SRB and (b) exo-PSH ITA, recorded at λ_exc_ of 780 nm.

Raman spectra of exo-PSHs of both strains (in their as-prepared, gel-like state) are shown in Figure 3.

The spectra are very similar to each other, showing main bands at the same wavenumbers, 2974, 2930, 2880, 2723, 1480, 1453, 1276, 1120, 1090, 1049, 881, and 435 cm^−1^, with small differences in the relative intensity of some bands. In the high-frequency region, both exo-PSH samples show three very strong bands at 2974, 2930 and 2880 cm^−1^, assigned to C–H stretching vibrations of PSHs [64,65]. Absorption bands in the region around 2900 cm^−1^ are common features in the vibrational spectra of carbohydrates and PSHs [64,65]. For comparison, the Raman spectrum of β-D-glucose (monomer for PSHs) shows bands at 2976, 2945, 2936 (shoulder), 2908, 2898, and 2880 cm^−1^ (shoulder) [64].

Both exo-PSH samples show a strong band at 1453 cm^−1^, accompanied by a weak band at ca. 1480 cm^−1^. Both bands are assigned to CH_2_ deformation vibrations with the contribution of O–C–H deformation, and the medium intensity band at 1276 cm^−1^ is attributed to the C–O–H bending mode in CH_2_OH groups. Similar bands appear in the Raman spectra of various PSHs [64], β-glucans [66,67] SPG, and curdlan [68].

The spectral region between 800 and 1200 cm^−1^ provides marker bands for identifying carbohydrates and differentiating between different PSHs (bands due to C–O–C, C–O, and C–C stretching vibrations and C–H bending vibrations) [67,69]. The region between ca. 1000 and 1200 cm^−1^ in the spectra of PSHs has been assigned to C–C and C–O stretching vibrations, with an important contribution arising from C–O stretching, which involves oxygen in the glycosidic C–O–C bond [70,71]. In that region, both exo-PSH samples show two medium-intensity bands, at 1090 cm^−1^ and 1049 cm^−1^. The band at ca. 1090 cm^−1^ is characteristic of β-glucans and is observed at similar positions in Raman spectra of several mixed β-(1→3)/β-(1→6)-D-glucan polymers, such as SPG, which consists of a backbone of β-(1→3)-glucose residues with β-(1→6)-linked glucose side chains at about every third residue [68,72], and the laminarin (β-(1→3)-linked polymer of D-glucose, with two or three branches per chain formed via β-(1→6)-linkages [70]. In the mentioned spectral region, the third (weak) band, assigned to the C–O–C stretching mode, is observed at ca. 1120 cm^−1^ for the exo-PSH ITA strain [71], but is hardly noticeable in the spectrum of exo-PSH SRB, where it appears as a shoulder. This feature indicates some fine structural differences between PSH products of the two investigated *S. commune* strains, possibly related to triple-helix formation and its effect on the vibration modes involving the glycosidic bond [70]. The Raman spectral region of 900–800 cm^−1^ is sensitive to configuration at the anomeric C1 position and is frequently used to differentiate between β- and α-glucans [66,69]. Raman marker bands of β-glucans, due to C–H bending vibrations, δ(C–H), appear in the ca. 905–885 cm^−1^ region, while α-glucans show characteristic bands in the ca. 865–835 cm^−1^ region [64,65,69]. Both exo-PSH samples show a very strong δ(C–H) band at 881 cm^−1^, which confirms the presence of β-glucan structure [65,69] and is seen at a similar position in the Raman spectrum of SPG [67]. In the Raman spectrum of curdlan, a predominantly linear β-(1→3)-D-glucan polymer band (which may have a few intra- or inter-chain (1→6)-linkages) [66,71] is observed at 891 cm^−1^ [71] and 886 cm^−1^ [68]. Based on the absence of bands in the 865–835 cm^−1^ region, it can be deduced that an α-anomeric configuration of glucan is not present in exo-PSH samples from either strain.

In the low-frequency region 600–350 cm^−1^, where skeletal bending vibrations of pyranose ring of PSHs appear [73], both exo-PSH samples show one band, at 435 cm^−1^. In the literature, the Raman band at ca. 423 cm^−1^ was commonly assigned to β-(1→3)-D-glucans [66,69] and attributed to mixed C–C, C–C–O, C–O–C, and C–C–H vibrations in D-glucose units [64]. Thus, the band at 435 cm^−1^ in the spectra of exo-PSH samples is related with β-(1→3)-D-glucan structures, and its higher wavenumber could be due to the influence of β-(1→6)-linked glucose side chains.

It can be concluded from the Raman spectra analysis that exo-PSH samples (both ITA and SRB) contain SPG type β-(1→3)/β-(1→6)-D-glucan PSH as a major product. Minor differences in the PSH molecular structure of the two strain products can be related to differences in triple-helix supramolecular structure formation. The weak band at 2723 cm^−1^ observed in Raman spectra of both samples is not characteristic of PSHs or SPG, but is commonly seen in the Raman spectra of fatty acids and lipids, e.g., dodecane and dodecanoic acid [74,75], thus indicating that relatively small amounts of such compounds are present in both exo-PSHs. The band observed at ca. 2720 cm^−1^ in the Raman spectrum of dodecane has been attributed to the symmetric stretching of the CH_3_ group or to the overtone of CH_2_ and CH_3_ deformation vibrations, which occur at about 1360 cm^−1^ [74]. The presence of other biological compounds, such as proteins, cannot be proven from Raman spectra, although it is not excluded that their bands could be covered by much stronger bands of PSHs and/or fluorescence background. Characteristic bands of proteins [69,76], such as the amide I band at ca. 1645–1685 cm^−1^, phenylalanine band at 1004 cm^−1^, or tyrosine band at about 850 cm^−1^ are not seen in Raman spectra, whereas the amide III band, expected in the 1200–1350 cm^−1^ region, could be masked by the PSH band at 1276 cm^−1^.

Raman spectra of endo-PSH samples (at λ_exc_ = 780 nm) were not usable because they contained a very strong, broad fluorescence signal that covered Raman scattering signals and could not be eliminated by photobleaching or satisfactorily corrected.

#### 3.2.5. FTIR Spectroscopy

**Figure 4:**
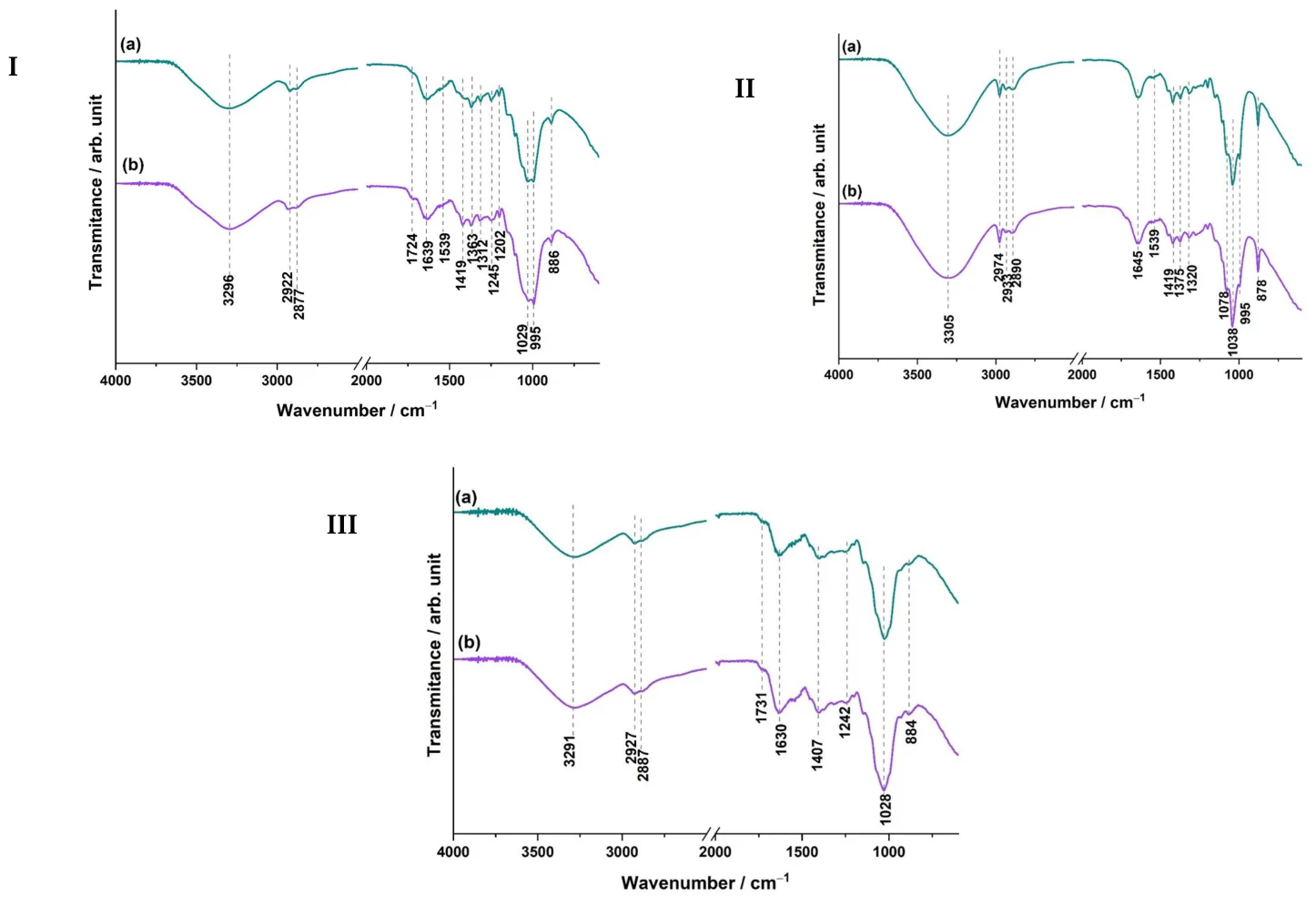
(**I**) FTIR 1 ATR-FTIR spectra of as-prepared samples: (a) exo-PSH SRB and (b) exo-PSH ITA. (**II**) FTIR 2 ATR-FTIR spectra of the samples: (a) exo-PSH SRB and (b) exo-PSH ITA after exposure to air at RT for 12 days. (**III**) ATR-FTIR spectra of as-prepared samples: (a) endo-PSH SRB and (b) endo-PSH ITA.

ATR-FTIR spectra of as-prepared exo-PSH samples are shown in Figure 4.

Most of their bands are consistent with those in the spectra of SPG reported in the literature, including commercial ones [72,77,78,79]. The spectra of exo-PSH SRB and exo-PSH ITA are mutually very similar, with small differences in relative intensities of some bands (which will be described below). Since it was noticed during preliminary investigations that during exposure to air at room temperature (RT), the sample volume decreases with loss of gel consistency and their spectra undergo certain changes, we recorded the spectra of exo-PSH samples after they had been left in the air for 12 days at RT (Figure 4II). This allowed us to identify which bands/functional groups are influenced by the release of associated water under air/RT conditions from exo-PSH and possibly by the breaking of hydrogen bonds involving water and metabolic products of *S. commune*.

A strong, broad band in the region 3600–3100 cm^−1^ with maximum at 3305 cm^−1^ is seen in the spectra of both as-prepared exo-PSH SRB and exo-PSH ITA samples (Figure 4I). It is attributed to O–H stretching vibrations, ν(O–H), of associated water in the samples, as well as to hydrogen-bonded ν(O–H) vibrations in PSHs, with possible contributions of ν(O–H) in phenolic compounds and N–H stretching of the *amide A* band in proteins [65,72,80]. Another band that can be attributed to associated water is seen at 1645 cm^−1^ in the spectra of both as-prepared EPSH samples, due to O–H deformation vibration [65]. These two bands are present at similar positions in the reported FTIR spectra of SPG produced from fungi [78,79], as well as commercial SPG [77]. The contribution of the amide I band of proteins and/or N–H bending in flavonoids to the band at 1645 cm^−1^ is possible [65,80]. After standing for 12 days in air at RT, the broad band in the region 3600–3100 cm^−1^ reduces its intensity and its maximum moves from 3305 to 3296 cm^−1^, whereas the band at 1645 cm^−1^ slightly reduces in intensity, becomes wider, and shifts to 1639 cm^−1^ (Figure 4II). These features indicate the release of associated water from exo-PSH samples when exposed to air at RT over a long period. It is known that SPG exists in water as a triple-helix structure and forms a weak, physical gel in cooled aqueous solution when its concentration is above a critical value, through the association of triple helices mediated by hydrogen bonds. Water molecules adopt different structures in the triple-helix state and form hydrogen bonds with SPG, playing an important role in gelation [81].

We also observed that several sharp bands seen in the spectra of the as-prepared exo-PSH samples disappeared (2974 cm^−1^) or weakened and shifted (from 1038 to 1029 cm^−1^, from 878 to 886 cm^−1^) in the spectra recorded upon standing in air at RT for 12 days (Figure 4II). These bands align well with the strongest FTIR bands of absolute ethanol (Figure S7). The mentioned spectral changes indicate the delayed release of residual ethanol from EPSH samples. Absolute ethanol was used to precipitate exo-PSH, and it was not completely removed during the exo-PSH isolation process. The most likely reason for the difficult removal of the ethanol solvent is its strong hydrogen-bonding O–H O interaction with water molecules and PSH, as all compounds have O–H bonds.

The bands at 2933 and 2890 cm^−1^ in the spectra of both as-prepared exo-PSHs shifted to 2922 and 2877 cm^−1^ upon 12 days of standing in air at RT. These bands are attributed to C–H stretching vibrations of PSHs [65] and correspond well with those of SPG from the literature [72].

In the FTIR spectral region 1200–750 cm^−1^, important for identification and structural characterization of PSHs [65,80], i.e., in two key sub-regions 1200–950 cm^−1^ (“sugar region”) and 950–750 cm^−1^ (“anomeric region,” commonly used to distinguish various types of glucans), the bands at around 1078 cm^–1^ (C–O–C stretching vibration), 1044 cm^–1^ (C–O stretching in C–OH group), and 890 cm^–1^ (C–H deformation vibration) were used as marker bands of fungal (1→3)(1→6)-β-D-glucans [66] and SPG [82]. These bands appear as very strong at 1078, 1038, 995, and 878 cm^−1^ in the spectra of as-prepared exo-PSH samples, and are observed at ca. 1078, 1029, 995 and 886 cm^−1^ after 12 days of standing in air at RT, confirming the presence of a β(1→3)(1→6)-D-glucan structure i.e., SPG PSH [72,78]. The band at 886 cm^−1^ corresponds well with the band found in the Raman spectra of both exo-PSH samples at 881 cm^−1^, but is stronger in Raman spectra (Figure 3) due to the higher sensitivity of Raman spectroscopy to non-polar/less polar bonds compared to FTIR spectroscopy.

One of the fine differences between the ATR-FTIR spectra of the two exo-PSH samples is a weak band at 1724 cm^−1^, which is noticeably stronger for exo-PSH ITA compared to exo-PSH SRB. This band is attributed to C=O stretching, ν(C=O), which can occur in biological compounds such as lipids, fatty acids, or phenolic acids [65,80]. This band becomes more visible after prolonged exposure of exo-PSH samples to air at RT (Figure 4I,II), which can be explained by breaking C=O…H–O hydrogen bonds with water, in which this band was originally included. Thus, FTIR spectra indicate that exo-PSH ITA has a slightly larger content of C=O group-containing biological compounds than exo-PSH SRB, confirming the results of Congo red (Figure S3) and SEM analysis (Figure 1). It should be noted that the relative intensities of marker bands indicate that the content of C=O group-containing compounds in both exo-PSHs is significantly smaller compared to the β-D-glucan SPG content. In the spectra of as-prepared exo-PSH, the band at 1419 cm^−1^ can be attributed to CH_2_ deformation vibration in PSHs, and the band at 1375 cm^−1^ (shifted to 1363 cm^−1^ after exposure to air at room T) corresponds to CH_2_ wagging (PSH, lipids) and CH_3_ symmetric bending (lipids) [65,80]. After exposure to air at RT, the band at 1419 cm^−1^ did not change position, but its intensity was higher for the exo-PSH ITA compared to exo-PSH SRB spectrum (Figure 4II). The weak band at 1539 cm^−1^ seen in the spectra of both exo-PSH samples (Figure 4I) can be attributed to C=C stretching vibration of aromatic rings in aromatic/phenolic compounds and to the amide II band of proteins [65], and is not noticeably changed after exposure of samples to air at RT (Figure 4II). The presence of proteins in both exo-PSH samples is also indicated by the band at 1320 cm^−1^, which corresponds to the amide III band [65] and is shifted to 1312 cm^−1^ upon prolonged exposure to air at RT. The band at 1245 cm^−1^ corresponds to O–H deformation vibration in PSHs [65,80], and becomes more pronounced and sharper for both exo-PSH samples after exposure to air at RT, most probably due to breaking of O–H…O hydrogen bonds with water. A weak band at 1202 cm^−1^ is seen for both exo-PSH samples, and has been attributed to C–O and C–O–C stretching related to the CH_2_OH group of the glucose side chains in β-glucans [71]. It is of similar intensity for both exo-PSH samples, and did not change upon exposure to air at RT.

ATR-FTIR spectra of endo-PSH SRB and ITA samples (in powder state) are shown in Figure 4III. There are no important differences between the spectra of these two samples. Regarding the position, shape, and relative intensity of the bands at about 3300, 2900, and 1640 cm^−1^, these spectra are more similar to the spectra of exo-PSH samples held in air at RT for 12 days than to those of as-prepared (gel-like) exo-PSH samples. In the spectra of endo-PSH, there is no sharp band at 2974 cm^−1^, which is seen in the as-prepared exo-PSH samples, supporting its assignment to residual ethanol. Main bands indicative of PSH/SPG are positioned at 3291, 2927, 2887, 1407, 1242, 1028, and 884 cm^−1^ for both endo-PSH samples. Their ATR-FTIR spectra also contain bands indicative of proteins, flavonoids, aromatic compounds (1630, 1539 cm^−1^), and lipids/fatty acids/phenolic acids (1731 cm^−1^, ν(C=O)), but the major component is PSH. Contrary to exo-PSH, there is no noticeable difference in the intensity of the weak band at 1731 cm^−1^ between endo-PSH SRB and endo-PSH ITA.

#### 3.2.6. TGA

**Figure 5:**
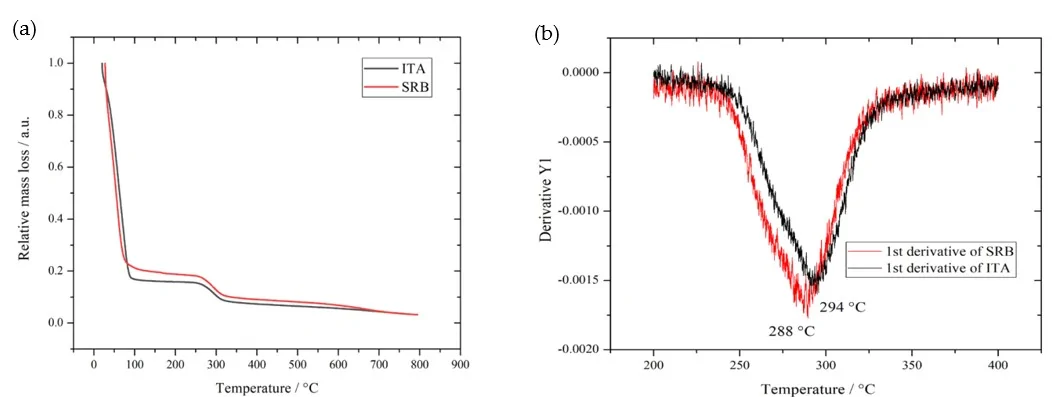

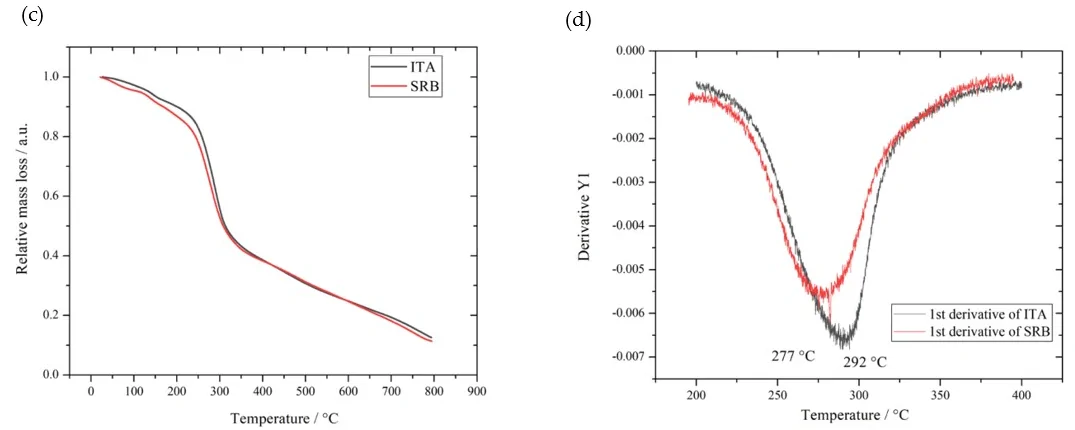
(**a**) Thermogravimetric analysis (TGA) and (**b**) derivative thermogravimetry (DTG) curves of exo-PSH from SRB and ITA strains; (**c**) TGA and (**d**) DTG curves of lyophilized endo-PSH samples from SRB and ITA strains.

The main feature of the examined gels (Figure 5a) is significant mass loss up to around 120 °C, 80% and 84% for exo-PSH SRB and exo-PSH ITA, respectively, which is to be expected.

This initial mass loss is generally attributed to the evaporation of physically bound water and other low-molecular-weight volatile compounds commonly associated with polysaccharide matrices [83]. There is a 6 °C difference (Figure 5b) between the onset of the structural transformation to a char. Exo-PSH SRB shows earlier transformation, which could indicate that its monostructure is less stable, while bimodal exo-PSH ITA structure is more robust, in accordance with Congo red results.

Lyophilized samples show only around 10% water loss up to 200 C as a result of the preparation procedure. Endo-PSH ITA losses adsorbed water with a few degrees’ delay over endo-PSH SRB (Figure 5c), which might point to differences in sample structure and consequent water binding. Degradation that occurs at 250–350 °C shows a larger difference of around 14 °C for powdered samples (Figure 5d) compared to gels, which further emphasizes the potential difference in structure of sample SRB and ITA. This initial mass loss is generally attributed to the evaporation of physically bound water and other low-molecular-weight volatile compounds commonly associated with polysaccharide matrices [83].

We hypothesize that higher water content in gels slightly influences the structure, so the differences in maximum charring temperature appear smaller (6 °C vs 14 °C). Above 400 °C for powder and 650 °C for gel samples, the samples transform into products that show identical temperature profiles.

### 3.3. Antioxidant Activity of Pea Extracts After Biopriming with PSHs Under Optimal and Drought Conditions

#### 3.3.1. Determination of *In Vitro* Enzymatic Activity

**Figure 6:**
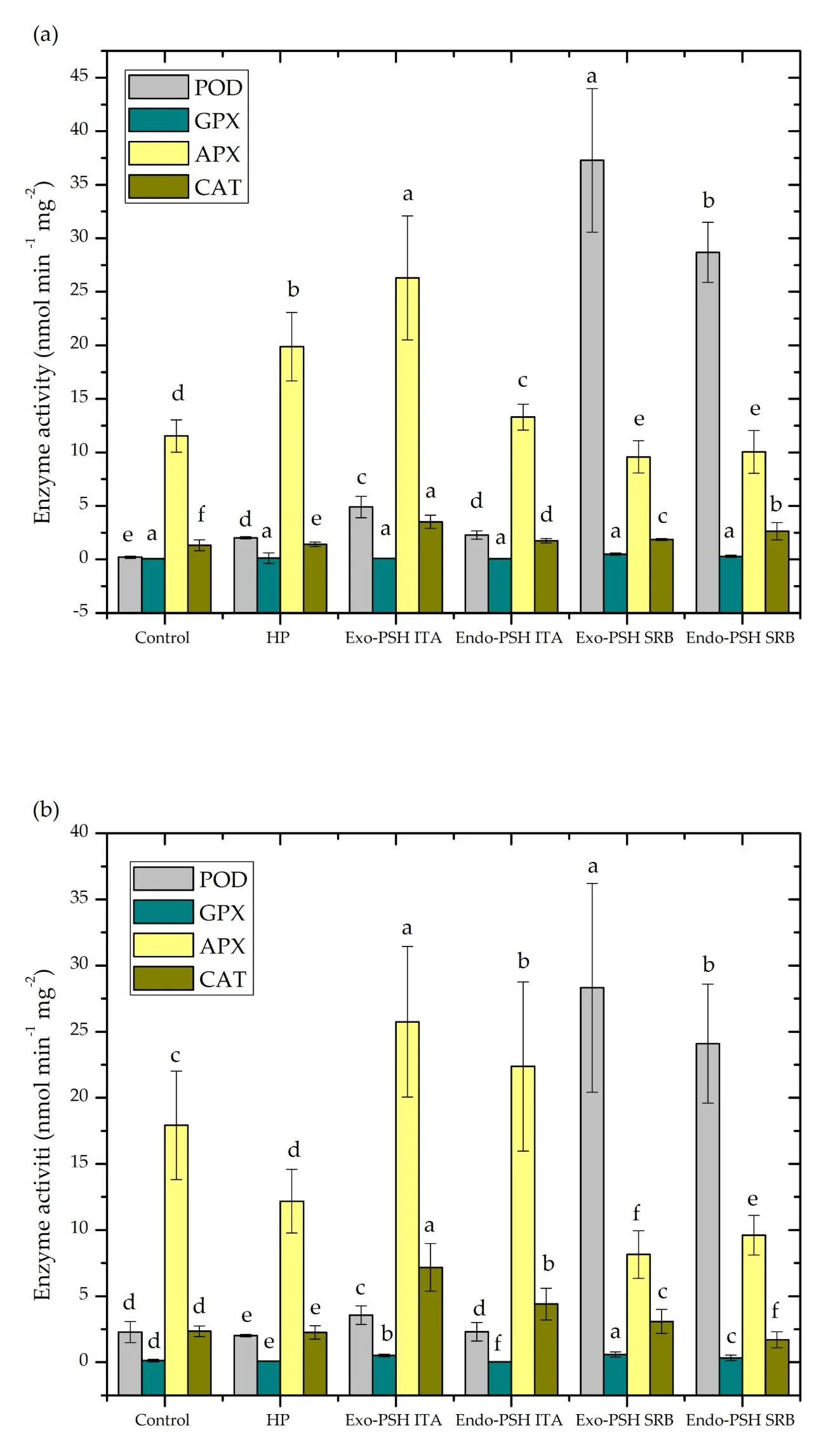
Enzyme activity of *Pisum sativum* L. extracts after biopriming with fungal PSHs under (**a**) optimal and (**b**) drought conditions. Statistical analysis was performed using two-way ANOVA followed by Tukey’s HSD post hoc test. Differences were considered statistically significant at *p* < 0.05. Different lowercase letters (a–f) indicate statistically significant differences among treatments, whereas bars sharing the same letter are not significantly different.

The contribution of PSHs isolated from the submerged culture of two strains in combating oxidative stress was assessed by investigating the activity levels of POD, GPX, APX, and CAT in fresh pea extracts (Figure 6).

Water-deficit conditions significantly increased the activity of all analyzed enzymes involved in reactive oxygen species (ROS) detoxification in the control group. This response may reflect the activation of the antioxidant enzymatic system in response to drought-induced oxidative conditions, suggesting that plants upregulate antioxidant enzyme production under drought stress as a protective mechanism to scavenge excess ROS and minimize cellular damage. These findings are consistent with the literature, which indicates that elevated antioxidant enzyme activity is a key component of the drought-stress response in plants. Moreover, certain genotypes, such as peas, wheat, and beans, have been shown to exhibit inherently higher levels of these enzymes, contributing to their greater drought tolerance [84,85].

Under both optimal and drought-stress conditions, PSH treatments also enhanced the activity of all tested enzymes. Exo-PSH isolated from both strains showed the most significant positive impact on enzyme activity, while exo-PSH SRB notably influenced POD and GPX activity, whereas exo-PSH from the ITA strain enhanced APX and CAT activity. However, exo-PSH SRB did not affect APX or CAT activity in pea, likely due to its pronounced effect on POD and GPX activity. These findings are consistent with studies indicating the positive impact of biopriming on enzyme activity [85,86]. Throughout evolution, plants have developed an antioxidant defense system, comprising enzymes like peroxidase and CAT, to participate in protection against oxidative damage induced by ROS [87]. ROS accumulation leads to oxidative stress, damaging proteins, DNA, and lipids. Thus, maintaining a balance between ROS production and elimination is critical [88]. Peroxidase and CAT play pivotal roles in the enzymatic processing of H_2_O_2_, a significant ROS. Elevated H_2_O_2_ levels are observed during plant exposure to abiotic stress such as drought. The increased activity of CAT and peroxidases in response to drought may contribute to the removal of H_2_O_2_ produced during stress and photorespiration. These findings are consistent with previous studies suggesting that enhanced plant stress tolerance is linked to improved oxidative-stress protection via an augmented antioxidant system [84,85,86,89]. Nevertheless, because ROS levels were not directly quantified in the present study, the observed changes in enzyme activity should be interpreted as an indication of modulation of the antioxidant response rather than direct evidence of enhanced ROS detoxification or reduced oxidative stress.

#### 3.3.2. Determination of Non-Enzymatic Antioxidant Activity

The antioxidant activity of MeOH pea extracts was tested by determining the ability of the extracts to neutralize DPPH and ABTS radicals in order to examine the effect of biopriming with fungal PSHs on the in vitro radical-scavenging activity of pea extracts (Figure S8). The strongest scavenger activity of DPPH and ABTS radicals was shown by plants whose seeds were subjected to treatment with an endo-PSH extract originating from the SRB strain of the mushroom *S. commune* both under optimal and stressful conditions. In addition, exo-PSH SRB affected the neutralization of both radicals in both pea root extracts and aerial part extracts. PSHs isolated from the ITA mushroom strain also had the effect of neutralizing the mentioned radicals, but to a much lesser extent. By comparing the scavenger activity of the roots and the aerial part of the pea, it is evident that the antioxidant activity is stronger in the aerial part, with the exception of neutralization of ABTS radicals after biopriming with exo-PSH SRB (33.55 ± 0.18 mg TE/g d.w.) and of DPPH radicals after HP (IC_50_ = 74.32 ± 29.71 µg/mL). This difference in activity can be explained by the different content of secondary metabolites, such as phenols and flavonoids, in the root and the shoot, considering that the aboveground part generally has a higher concentration of compounds that are known to affect antioxidant activity [90].

To the best of our knowledge, there is no literature comparing DPPH and ABTS RSC activity following biopriming of peas with PSHs. However, in contrast, research on pea seed MeOH extract demonstrated significantly lower DPPH radical neutralization, with an IC_50_ value of 650 ± 0.05 µg/mL [91], nearly four times weaker than the control (IC_50_ = 168.95 ± 4.05 µg/mL) and six times weaker than the activity observed after biopriming with endo-PSH SRB (IC_50_ = 109.93 ± 2.30 µg/mL). Studies by Tančić-Živanov et al. [92] illustrated enhanced ABTS radical neutralization in pepper following biopriming with *Trichoderma* spp., while Lee et al. [93] suggested that hydropriming of cabbage seeds boosts ABTS and DPPH radical neutralization. These findings indicate that PSH biopriming can modulate the in vitro radical-scavenging activity of pea extracts; however, they should not be considered direct evidence of enhanced physiological antioxidant defense in the living plant.

The results of the FRAP test show that under optimal conditions, PSH treatments did not significantly affect the reduction potential, considering that the activity is similar to the values obtained in the control (Figure S8). A similar situation was recorded under conditions of stress, i.e., after a drought. However, it is important to note that in both tested conditions, the strongest reduction potential was shown by pea extracts whose seeds were treated with endo-PSH SRB extract. The weak reduction potential can be attributed to the use of MeOH as the extraction solvent, as previous studies have shown that solvent with similar polarity (70% ethanol) is more effective for FRAP assays in peas [91,94].

OH radical-scavenging capacity and inhibition of lipid peroxidation were investigated in MeOH extracts of the aerial part of pea in order to evaluate the in vitro antioxidant-related properties of the extracts following biopriming with fungal PSHs under optimal and drought-stress conditions. In terms of the neutralization of OH radicals under optimal conditions, pea extracts after HP showed no activity, while the control showed weak activity (Figure S9).

On the other hand, the highest OH radical-scavenging capacity was achieved in pea extracts whose seeds were treated with endo-PSH ITA (IC_25_ = 5.91 ± 0.01 µg/mL), while under drought the greatest effect was achieved after biopriming with endo-PSH SRB (IC_25_ = 5.00 ± 0.01 µg/mL). The increased OH activity observed under stress conditions may indicate an enhanced antioxidant response under drought conditions, consistent with findings reported in previous studies [86,87,95].

Lipid peroxidation of membrane lipids leads to cell membrane damage, which causes content leakage and leads to rapid desiccation and cell death, as a result of which inhibition of this process serves as a reliable indicator of plant antioxidant defense during abiotic stress [96]. In Figure S9, it can be clearly seen that during drought conditions, the inhibition of lipid peroxidation increased in all samples, including the control. The inhibitory effect of biopriming with PSHs isolated from the ITA strain on peroxidation was much weaker compared to the SRB strain. Moreover, the strongest inhibition was exhibited by the pea extract whose seeds were treated with endo-PSH SRB extract, both in optimal (IC_25_ = 61.86 ± 4.28 µg/mL) and in drought conditions (IC_25_ = 59.91 ± 5.34 µg/mL).

The results indicate that drought stress may led to increased membrane damage, as evidenced by elevated electrolyte leakage and LP. However, biopriming proved beneficial in all treated groups, significantly reducing electrolyte leakage and enhancing free radical neutralization compared to untreated controls. This effect is likely associated with changes in membrane stability and repair processes following priming [97], although the underlying mechanism was not directly investigated in the present study.

Moreover, the antioxidant activity of pea seedling extracts was markedly enhanced following biopriming with *S. commune*-derived PSHs, with a notably stronger effect observed from PSHs isolated from the SRB strain. Specifically, endo-PSH SRB demonstrated a more pronounced effect on non-enzymatic antioxidant activity, while exo-PSH SRB more effectively stimulated enzymatic antioxidant responses.

In addition to modulating oxidative-stress responses, biopriming probably contributed to drought tolerance in peas. This improvement is likely due to the formation of a hydrophilic biofilm layer by fungal PSHs during the priming process, which may contribute to increased water retention and protection under water-deficit conditions. Taken together, these results demonstrate that PSH biopriming altered several enzymatic and in vitro antioxidant-related parameters in pea seedlings. However, because direct ROS measurements were not performed, these findings alone do not establish whether PSH treatment reduced oxidative stress or directly enhanced drought tolerance.

Overall, a clear strain-dependent pattern was observed, where exo-PSHs from SRB more prominently modulated enzymatic antioxidant activity, whereas SRB-derived PSHs, particularly endo-PSHs, exerted a stronger effect on non-enzymatic radical-scavenging activity, indicating partially distinct effects of action between ITA and SRB treatments. The presence of β-glycosidic linkages and variations in the degree of branching may contribute to differences in conformational flexibility and biological accessibility of PSHs, which in turn could explain the observed variation in antioxidant activity and biostimulant-related effects between the investigated strains.

### 3.4. Chemical Characterization of Pea Extracts After Biopriming with Fungal PSHs Under Drought Conditions

#### 3.4.1. Total Chlorophyll Content

To assess the effect of fungal PSHs on photosynthetic pigment content under optimal and drought-stress conditions, the levels of photosynthetic pigments, including chlorophyll a, chlorophyll b, and total chlorophyll (a + b), were determined in pea leaves (Figure S10). In optimal conditions, biopriming with PSHs did not significantly affect the increase in pigment content in leaves, which agrees with the results obtained in the work of Miljaković et al. [98] after osmo- and hormopriming of soybean seeds. However, water deficiency affected the reduction of photosynthetic pigments, which is in accordance with a previous study on *Triticum aestivum* under nitrogen and water-deficiency conditions [99]. Moreover, proteomic analyses revealed that water deficiency disrupted the Calvin cycle by altering electron transfer and the ATP/NADPH ratio and reduced levels of CURT1 proteins suggested impaired thylakoid architecture [98]. The harmful effect of drought most likely led to a reduction in photosynthesis and thus to a reduction in chlorophyll content, as previously proven in beans, cabbage, alfalfa, and soybeans [98,100,101,102]. However, the obtained results may indicate greater protection of measured photosynthetic pigments from the stress effects caused by water deficit in plants treated with water (HP) or PSH extract (biopriming) under drought stress. Biopriming with endo-PSH ITA significantly increased the total chlorophyll content by 26.12% compared to the control and by 27.85% compared to HP. An increase in the number of chloroplasts in leaves or a moderate number of toxic ions in leaves that cannot lead to early leaf senescence or chlorophyll degradation may be the source of the increase in chlorophyll content in plants obtained after seed biopriming [97]. Overall, endo-PSH ITA showed the greatest influence on the content of chlorophyll a and b and on the total content of these pigments in both optimal conditions and water-deficit conditions, while PSHs originating from the SRB fungal strain showed the weakest influence on the measured photosynthetic pigments of pea leaves.

#### 3.4.2. Total Protein Content (TPR) of Pea Extracts

**Figure 7:**
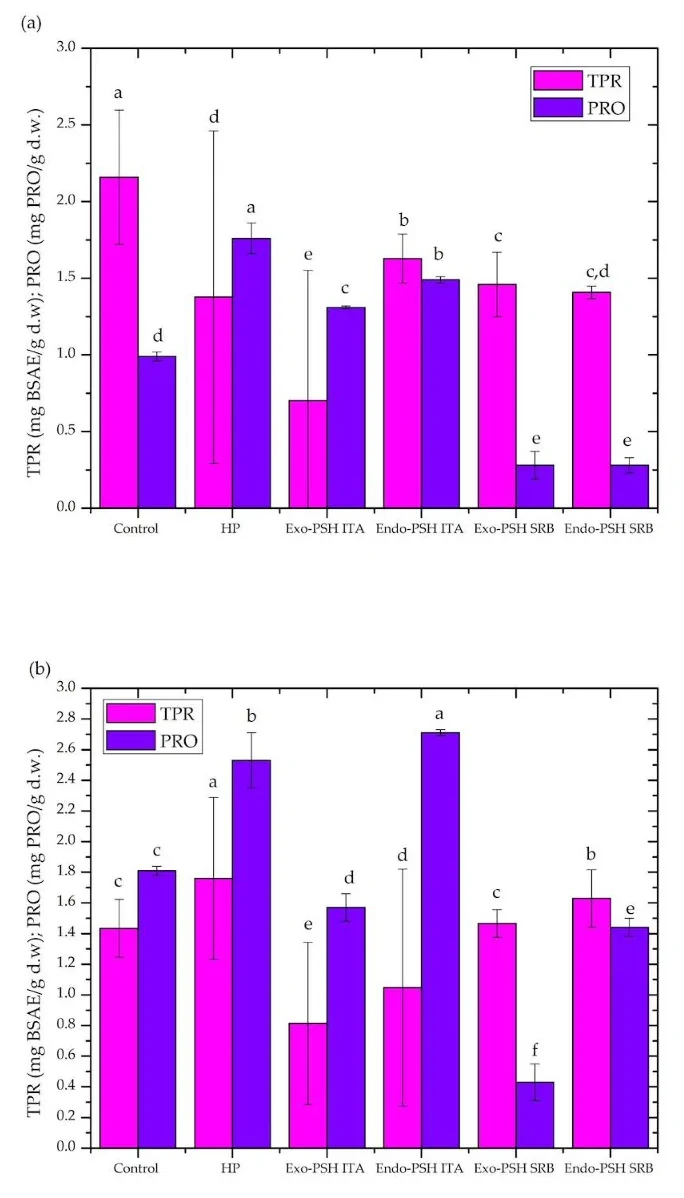
Total protein content (TPR) and total proline content (PRO) of *Pisum sativum* L. extracts after biopriming with fungal polysaccharides under (**a**) optimal and (**b**) drought conditions. Statistical analysis was performed using two-way ANOVA followed by Tukey’s HSD post hoc test. Differences were considered statistically significant at *p* < 0.05. Different lowercase letters (a–f) indicate statistically significant differences among treatments, whereas bars sharing the same letter are not significantly different.

Results of TPR in pea extracts after biopriming with fungal PSHs are presented in Figure 7.

The highest TPR was determined in the control extract and the endo-PSH ITA extract under optimal conditions (2.16 ± 0.44 mg/mL and 1.63 ± 0.16 mg/mL, respectively). Additionally, the treatment with PSHs originating from the SRB strain under water-deficit conditions had a greater effect on the increase in protein content compared to the treatment with PSHs from the ITA strain. On the other hand, by comparing the influence of the type of PSH on TPR, the results indicate that a higher TPR was determined after biopriming with endo-PSH both in optimal conditions and in conditions of water deficit compared to exo-PSH.

The results agree with previous studies where TPR under drought conditions in peas, corn and barley was reduced compared to optimal conditions [103,104,105]. In addition, biopriming with PSHs isolated from *C. vulgaris* significantly increased the TPR of wheat and bean extracts under optimal conditions [106], in contrast to the study of tomatoes in drought conditions, where higher TPR was recorded [107]. However, in the same work, treatment of tomato seeds with *T. harzianum* had a positive effect on TPR [107], while in our study, the positive influence of biopriming with endo-PSH SRB on TPR in pea extracts was confirmed. This contradiction can be explained by the fact that a plant exposed to drought conditions can respond in different ways, depending on the duration of the stress, the degree of stress, the plant genotype, and the applied treatment [108]. In response to a lack of water, a plant can experience cell damage and metabolic disorders, which can lead to protein degradation and inhibition of protein synthesis due to the allocation of resources to basic functions, such as turgor, water transport and energy production [108]. Exposure to drought stress leads to ROS accumulation, which causes loss of turgor due to membrane damage from lipid peroxidation and further cytoplasmic leakage [109]. However, biopriming improves defense mechanisms, selective absorption, membrane stability and stress tolerance [109]. Additionally, biopriming can have a positive effect on TPR, which is mainly attributed to the activation of specific mechanisms of synthesis and expression of certain proteins [110]. Furthermore, PSH treatment most likely enabled the plant to use soluble PSHs as a source of carbon and nitrogen [106], as a result of which a higher TPR was recorded in pea extracts whose seeds were subjected to biopriming.

#### 3.4.3. Total Proline Content (PRO) of Pea Extracts

The rapid accumulation of free PRO in plant cells is commonly associated with plant responses to stressful conditions [111], as a result of which the PRO of pea extracts was determined after biopriming with PSHs isolated from *S. commune* (Figure 7). In optimal conditions, the highest PRO was recorded after HP (1.76 ± 0.10 mg eq. PRO/g d.w.). However, observing the influence of biopriming on PRO in optimal conditions and in drought, the highest content was determined in extracts treated with endo-PSH ITA (1.49 ± 0.02 and 2.71 ± 0.02 mg eq. PRO/g d.w., respectively) given that the concentration of PRO was 1.5 times higher compared to the control (0.99 ± 0.03 and 1.81 ± 0.02 mg eq. PRO/g d.w., respectively).

The main functions of PRO include protection of macromolecules from denaturation and carbon and nitrogen reserves, and it may contribute to osmotic adjustment, osmoprotection, redox regulation and maintenance of cellular homeostasis [111,112]. It has also been established that PRO protects metabolic processes under stressful conditions by replacing water and thereby contributing to the stability of important cellular structures [107,112]. The obtained results are consistent with previous studies reporting a positive association between PRO accumulation and plant tolerance to stressful conditions [105,113]. This is in agreement with the higher PRO observed under water-deficit conditions. Moreover, it was confirmed that biopriming of pea seeds with *T. angustifolia* leaf extracts led to higher PRO accumulation [97], even in relation to the plant’s response to salinity-stress conditions, while Mona et al. [107] indicated that treatment of tomato seeds with *T. harzianum* has a positive effect on PRO under water-deficit conditions. However, some authors question the ability of PRO to directly neutralize ROS [114], while others argue that it may contribute to maintaining the redox balance in chloroplasts and mitochondria [113,115]. This view is supported by Vuksanović et al. [116], who demonstrated that drought stress induced by polyethylene glycol (PEG 6000) significantly increased PRO accumulation in all tested white poplar clones, and that PRO was moderately to strongly correlated with antioxidant activity (FRAP and ABTS). Their findings underscore the potential role of PRO not only as a compatible osmolyte, but also as an antioxidant and a signaling molecule that helps maintain cellular homeostasis under drought stress [116].

#### 3.4.4. Total Phenolic (TPC) and Total Flavonoid Content (TFC) of Pea Extracts

TPC and TFC were determined in extracts of the aerial part and roots of peas treated with PSHs isolated from the ITA and SRB strains of *S. commune* (Table S3). The study showed that the pea root extract had the highest TPC after HP (45.79 ± 3.45 mg GAE/g d.w.), whereas the control root extract had the highest TFC under optimal conditions (2.21 ± 0.11 mg QE/g d.w.). Conversely, in drought conditions, the highest TPC and TFC were found in extracts from both the roots and aerial parts of peas after biopriming with endo-PSH. In summary, elevated TPC and TFC were observed in aerial part extracts relative to root extracts in both conditions, with endo-PSH biopriming demonstrating the most pronounced effect on augmenting their levels; notably, endo-PSH SRB treatment proved most efficient in enhancing TPC under both conditions and TFC concentration under optimal conditions, while endo-PSH ITA treatment exerted the greatest influence on increasing TFC under drought stress.

Increased accumulation of TPC and TFC was recorded in response to biopriming in both tested conditions, which agrees with previous research showing that biopriming pea seeds with *T. angustifolia* extracts positively affected the total content of these compounds [97] mentioned. Dalal et al. [106] proved that biopriming of wheat and bean seeds with PSHs isolated from *C. vulgaris* stimulated a higher accumulation of TPC. A connection between stimulation of the initial growth of peas and increased TPC after treatment of seeds with *Trichoderma* extracts was also indicated, which agrees with our results [117]. This further indicates that increased TPC may contribute to the seed vigor response by stimulating antioxidant activity as shown in our previously published research [14].

However, certain literature suggests that under stressful conditions, plants tend to exhibit higher TPC and TFC, as increased oxidative stress prompts their elevated production [111], although this was not observed in the specific extracts analyzed in this study (Table S3). However, the results obtained agree with the results of Mona et al. [107], where TPC and TFC were reduced under drought conditions, while the treatment of tomato seeds with *T. harzianum* affected the increased accumulation of these compounds. Moreover, the increased synthesis of phenols and flavonoids after treatment with PSHs originating from the SRB strain was associated with improved initial growth of peas under drought conditions and may contribute to may contribute to the antioxidant-related properties of the extracts. However, the present data do not allow us to conclude that these compounds directly protected the plant against oxidative stress through ROS elimination. This supports the potential role of biopriming by fungal PSHs as a trigger of stress-responsive metabolic changes under drought-stress conditions in peas [14]. The higher accumulation of TPC and TFC in optimal conditions in control and HP extracts compared to drought-stress conditions can be explained by the fact that TPC can be influenced by the type of solvent, the method of extraction, and the type of extract (root, aerial part, seed coat, etc.) [118]. In addition, it was determined that the variation of the TPC and TFC in pea extracts is a consequence of different genotypes, i.e., the different color and shape of the seed coat, considering that it was determined that hollow and round seed coats, as well as dark-colored seeds, have a higher TPC [118].

#### 3.4.5. Chemical Profile of Pea Extracts Determined by LC–MS/MS

Among the 62 compounds examined, 17 phenolic compounds were quantified through the LC–MS/MS procedure by analyzing MeOH extracts of peas after treatment with fungal PSHs under both optimal and drought-stress conditions (Table 2).

The most abundant compound was the flavonoid liquiritigenin (61.92 ng/mg), detected in the extract of pea roots treated with endo-PSH SRB under optimal conditions, while in drought conditions the highest content of protocatechuic acid (60.15 ng/mg) was recorded in the extract of the aerial part of pea treated with exo-PSH ITA. Hydroxycinnamic acid, *p*-coumaric acid and the flavonoid naringenin were detected in all tested samples, but the higher content of these compounds was present in optimal conditions. The same trend was observed by comparing the content of *p*-coumaric acid and naringenin in relation to the mushroom strain (SRB/ITA) with which the plant was treated, considering that pea extracts after biopriming the seeds with PSHs originating from the SRB strain are richer in the mentioned compounds in both tested conditions. The content of naringenin in optimal conditions in the ITA strain is 11.56 ng/mg, and in the SRB strain 13.76 ng/mg, while in drought conditions it is similar to the amount detected in optimal conditions (ITA, 9.35 ng/mg; SRB, 11.72 ng/mg). Conversely, *p*-coumaric acid content was reduced by almost 50% in both strains in drought (ITA, 39.31 ng/mg; SRB 75.5 ng/mg).

Secondary metabolites play a major role in plant adaptation to environmental conditions and avoidance of stressful conditions, as a result of which the plant accumulates various compounds [97,119]. In this regard, the different accumulation of selected secondary metabolites is a consequence of the different types of analyzed extracts (root, aerial part), different treatments (biopriming), as well as the conditions in which the plant grew, given that different content is present in optimal and drought conditions, which is also the case with the results of TPC and TFC. The results obtained are in agreement with previous research, where priming of pea seeds with aqueous extracts of *T. angustifolia* leaves stimulated different production of secondary metabolites, including polyphenols and flavonoids, under optimal and stress conditions (salinity) [97].

The diverse enzymatic and non-enzymatic antioxidant activities observed in differently treated pea extracts suggest that secondary metabolites, such as catechins, flavones, and flavonols, may contribute to the observed antioxidant-related properties, as their levels were associated with the neutralization of free radicals [120,121]. Our results indicate that phenolic acids may be responsible for enzymatic antioxidant activity, since the best enzymatic activity (CAT and APX) was shown by the plant extract treated with exo-PSH ITA (3.52 and 26.3 nmol min^−1^ mg^−2^ under optimal conditions and 7.17 and 25.75 nmol min^−1^ mg^−2^ under drought conditions, respectively), in which a larger amount of protocatechuic acid was detected. Moreover, previous research shows that protocatechuic acid is very effective in preventing oxidative stress and may promote CAT and APX activity [122,123]. Conversely, for the strong neutralization of tested free radicals and reduction potential by pea extracts treated with SRB strain PSHs, especially endo-PSH, the main role is most likely played by the detected flavones, isoflavones, flavonones and flavonoids, whose content is the most represented in the mentioned extracts. More specifically, for the inhibition of lipid peroxidation, neutralization of ABTS and OH radicals, and the reduction potential of pea extracts subjected to biopriming with endo-PSH SRB, naringenin and naringin probably played the greatest role. These results are supported by previous research [124], while Stanisavljević et al. [125] reported a strong positive correlation between TPC and DPPH radical-scavenging activity (r = 0.968) in pea seed coat extracts, with gallic acid, epigallocatechin, naringenin, and apigenin showing significant positive associations with DPPH activity, further supporting the contribution of phenolic compounds to the antioxidant capacity of pea extracts. On the other hand, liquiritigenin has also been reported to exhibit DPPH radical-scavenging activity, with inhibition exceeding 65% under the tested conditions [126]. This finding supports the potential contribution of liquiritigenin, the most abundant flavonoid detected in pea extracts treated with endo-PSH SRB, to the antioxidant activity observed in the present study. Liquiritigenin and protocatechuic acid, the most abundant compounds identified under SRB and ITA treatments, appear to play key strain-dependent roles in modulating antioxidant-related responses. Their differential accumulation under optimal and drought conditions further coincided with the observed divergence between ITA- and SRB-derived PSHs in the measured biochemical parameters of pea seedlings. Importantly, these associations should not be interpreted as evidence of direct causality, as the present study did not experimentally isolate the contribution of individual phenolic compounds to the measured responses.

### 3.5. Correlation and Principal Component Analysis (PCA)

Pearson correlation analysis highlighted positive correlations between individual phenolic compounds (hormomonetin, naringine, ferulic acid, liquiritigenin and isoliquiritigenin) and ABTS and POD/GPX antioxidant parameters (Figure S11a). Under drought conditions, Pearson correlation analysis revealed strong positive associations between POD activity and the phenolic compounds naringin, hormonomethin, and diosmetin, indicating that the phenolic profile was closely related to enzymatic antioxidant responses under drought stress (Figure S11b). These findings are interpreted as associations/correlations rather than evidence of causality.

Quantified compounds under optimal conditions were also subjected to statistical PCA analysis for enzymatic and non-enzymatic antioxidant activity (Figure S12). In the case of optimal conditions, the variability of the first axis is 69.00%, while the variability of the second axis is 1.46% (Figure S12a). Furthermore, the clustering of most quantified phenolic acids in the positive part of the first axis is clearly visible, while most flavonoids are present in the negative part. The results indicate that enzymatic activities in pea extracts are positively associated with the levels of flavonoids and phenolic acids, particularly liquiritigenin and ferulic acid. This observation is in line with the findings of Sharma et al. [127], who reported that ferulic acid can induce POD activity and contribute to the reduction of H_2_O_2_ levels in wheat. Enzyme activity of APX and CAT is presented in the I quadrant, where pea extracts treated with exo-PSH ITA are grouped, suggesting that mostly flavonoids (apigenin, vitexin, amentoflavone etc.) are responsible for the stimulation of these enzymes. Regarding neutralization of free radicals, most quantified phenolic compounds were associated with antioxidant activity, particularly prominent in endo-PSH SRB extracts, implying a role of newly detected flavonoids and phenolic acids in these activities. Furthermore, OH radical-scavenging ability was mostly associated with detected flavonoids, especially after treatment with exo-PSH ITA.

The total variance for antioxidant activity under drought was 96.94% (Figure S12b). All secondary metabolites are separated in the positive part in relation to PC2, suggesting a positive correlation with enzymatic and non-enzymatic activity. Furthermore, the separation of the observed compounds could suggest the importance of flavonoids like liquiritigenin and naringenin in neutralizing radicals [124,126]. Compounds such as amentoflavone and protocatechuic acid also show a positive correlation with reduction potential, consistent with previous research findings [128,129]. In contrast, DPPH-scavenging ability is grouped separately, suggesting that other compounds may be responsible for neutralizing this radical. These multivariate relationships reinforce the strain-specific metabolic and biochemical responses induced by PSH treatments, linking distinct phenolic profiles with differential modulation of antioxidant defense pathways under both optimal and drought conditions.

### 3.6. Limitations of the Study

Several limitations of the present study should be acknowledged. First, seed biopriming was evaluated using a single PSH concentration (1%), which limits the assessment of concentration-dependent effects and prevents identification of the optimal treatment concentration. Second, although the biological effects of fungal PSHs were demonstrated, their structural characterization did not include detailed determination of monosaccharide composition, molecular weight distribution, or purity. The absence of a detailed purity assessment also means that residual proteinaceous or phenolic impurities cannot be excluded as potential contributors to the observed biological activities, which therefore cannot be attributed exclusively to the polysaccharide fraction. Therefore, the obtained characterization data should be interpreted as providing complementary structural evidence rather than a complete description of PSH composition and architecture. Furthermore, the LC–MS/MS analysis was focused on the quantitative profiling of selected secondary metabolites, including phenolic compounds, flavonoids, and lignans, and therefore does not represent a comprehensive characterization of all bioactive constituents that may contribute to the observed effects. Finally, the experiments were performed under controlled conditions using a single plant species, and therefore the transferability of the observed responses to other crops and field conditions remains to be established.

Future studies should address these limitations by evaluating a broader range of PSH concentrations, performing more detailed structural characterization of the polysaccharide preparations, and integrating metabolomic and molecular approaches to elucidate the mechanisms underlying PSH-mediated plant responses. Validation across different crop species and under field or semi-field conditions would further clarify the practical potential of fungal PSHs as biopriming agents.

## 4. Conclusions

This study demonstrated for the first time that PSHs obtained from two *S. commune* strains can modulate several antioxidant-related and biochemical parameters in *Pisum sativum* L. seedlings following biopriming. The observed responses differed according to the *S. commune* strain and PSH type, indicating variation in the effects of the tested preparations. The exo- and endo-PSHs from the SRB strain exhibited more pronounced effects on the antioxidant-related parameters evaluated in the present study, including the in vitro radical-scavenging and -reducing capacities of the extracts. In contrast, endo-PSHs from the ITA strain were associated with higher chlorophyll and proline content, indicating their differential effects on the biochemical parameters measured in the seedlings.

LC–MS/MS profiling and multivariate analysis identified several phenolic compounds, including liquiritigenin, ferulic acid, and protocatechuic acid, that could be associated with the observed response patterns. However, these associations should be considered exploratory and do not establish direct causal relationships between individual compounds and the measured biological responses.

Overall, the present findings indicate that PSHs from different *S. commune* strains can differentially modulate antioxidant-related and biochemical parameters in pea seedlings and may have potential as fungus-derived preparations for seed biopriming. However, the present experiment does not directly demonstrate enhanced drought tolerance or resistance in laboratory conditions, improved photosynthetic efficiency, or specific mechanisms of action. Further studies under controlled drought-stress conditions, including direct measurements of physiological and oxidative-stress parameters, as well as molecular and field-level investigations, are needed to determine whether these preparations can improve plant stress tolerance and to elucidate the mechanisms underlying the observed responses.

## Figures and Tables

**Table 1:** Overview of the experimental design, showing all combinations of growth conditions, priming treatments, PSH origin, PSH type, and corresponding experimental groups.

Growth Condition	Priming Treatment	PSH Origin	PSH Type	Group
Optimal	No priming	-	-	Control
	Hydropriming	-	-	HP
	Biopriming	ITA	Exo-PSH	ITA
	Biopriming	ITA	Endo-PSH	ITA
	Biopriming	SRB	Exo-PSH	SRB
	Biopriming	SRB	Endo-PSH	SRB
Drought	No priming	-	-	Control
	Hydropriming	-	-	HP
	Biopriming	ITA	Exo-PSH	ITA
	Biopriming	ITA	Endo-PSH	ITA
	Biopriming	SRB	Exo-PSH	SRB
	Biopriming	SRB	Endo-PSH	SRB

**Table 2:** Concentrations of selected compounds determined by LC–MS/MS in the tested MeOH extracts of pea after biopriming with polysaccharides derived from *S. commune* (ng/mg dry weight).

Treatment/extract	Protocatechuic acid	*p*—Coumaric acid	Ferulic acid	Apigenin	Genistein	Naringenin	Apigenin-7-*O*-Glc	Vitexin	Kaempferol-3-*О*-Glc	Amentoflavone	Apiin	Liquiritgenin	Isoliquiritegnin	Formononetin	Diosmetin		Ursolic acid	Naringin
Optimal conditions																		
Control, root	<24.50	10.85	<12	<6	<6	3.24	<0.75	<6	<12	<24.50	<6	n.a.	n.a.	n.a.	n.a.		n.a.	n.a.
Control, aerial part	<24.50	10.23	17.10	<6	<6	1.63	<0.75	<6	<12	<24.50	<6	n.a.	n.a.	n.a.	n.a.		n.a.	n.a.
HP, root	<24.50	10.64	<12	<6	<6	1.71	<0.75	<6	<12	<24.50	<6	n.a.	n.a.	n.a.	n.a.		n.a.	n.a.
HP, aerial part	<24.50	27.13	17.83	<6	<6	2.40	<0.75	<6	<12	<24.50	<6	n.a.	n.a.	n.a.	n.a.		n.a.	n.a.
Exo-PSH ITA, root	<24.50	7.45	<12	<6	<6	2.27	<0.75	<6	<12	48.73	<6	n.a.	n.a.	n.a.	n.a.		n.a.	n.a.
Exo-PSH ITA, aerial part	<24.50	24.68	21.69	<6	<6	3.05	<0.75	<6	<12	<24.50	<6	n.a.	n.a.	n.a.	n.a.		n.a.	n.a.
Endo-PSH ITA, root	<24.50	7.31	<12	<6	<6	4.14	<0.75	<6	<12	<24.50	<6	n.a.	n.a.	n.a.	n.a.		n.a.	n.a.
Endo-PSH ITA, aerial part	<24.50	24.56	25.31	<6	<6	2.10	<0.75	<0.75	<12	<24.50	<6	n.a.	n.a.	n.a.	n.a.		n.a.	n.a.
Exo-PSH SRB, root	<24.50	29.02	<49	<1.5	<3	1.90	<0.75	1.03	4.40	<12	<0.75	30.71	6.81	5.57	<1.5		18.86	1.64
Exo-PSH SRB, aerial part	<24.50	30.27	<49	1.60	<3	2.17	1.00	3.45	6.22	<12	1.19	28.64	6.71	6.36	2.32		28.24	11.77
Endo-PSH SRB, root	29.75	46.66	<49	<1.5	4.49	6.26	3.18	2.73	13.09	<12	2.72	61.92	15.43	25.47	<1.5		61.68	2.06
Endo-PSH SRB, aerial part	<24.50	16.36	<49	<1.5	3.06	3.43	2.77	<0.75	10.75	<12	2.46	19.78	5.96	7.54	<1.5		18.24	4.46
Drought																		
Control, root	<24.50	10.14	14.45	<6	<6	2.14	<0.75	<6	<12	<24.50	<6	n.a.	n.a.	n.a.	n.a.	n.a.	n.a.	n.a.
Control, aerial part	<24.50	19.96	29.42	<6	<6	1.59	<0.75	<6	<12	<24.50	<6	n.a.	n.a.	n.a.	n.a.	n.a.	n.a.	n.a.
HP, root	<24.50	7.13	17.59	<6	<6	2.14	<0.75	<6	<12	<24.50	<6	n.a.	n.a.	n.a.	n.a.	n.a.	n.a.	n.a.
HP, aerial part	<24.50	15.01	21.45	<6	<6	1.76	<0.75	<6	<12	<24.50	<6	n.a.	n.a.	n.a.	n.a.	n.a.	n.a.	n.a.
Exo-PSH ITA, root	<24.50	8.22	20.97	<6	<6	2.86	<0.75	<6	<12	<24.50	<6	n.a.	n.a.	n.a.	n.a.	n.a.	n.a.	n.a.
Exo-PSH ITA, aerial part	60.15	9.19	<12	<6	<6	2.45	<0.75	<6	<12	<24.50	<6	n.a.	n.a.	n.a.	n.a.	n.a.	n.a.	n.a.
Endo-PSH ITA, root	<24.50	7.25	<12	<6	<6	2.49	<0.75	<6	<12	<24.50	<6	n.a.	n.a.	n.a.	n.a.	n.a.	n.a.	n.a.
Endo-PSH ITA, aerial part	<24.50	14.65	17.59	<6	<6	1.55	<0.75	<6	<12	<24.50	<6	n.a.	n.a.	n.a.	n.a.	n.a.	n.a.	n.a.
Exo-PSH SRB, root	<24.50	12.68	<49	<1.5	<3	1.63	0.94	1.05	3.37	<12	1.11	20.08	3.44	4.46	<1.5		19.00	4.,80
Exo-PSH SRB, aerial part	<24.50	18.85	<49	<1.5	<3	1.90	1.06	1.31	4.83	<12	1.48	19.38	4.92	3.30	<1.5		11.30	6.46
Endo-PSH SRB, root	<24.50	19.34	<49	<1.5	<3	3.80	3.47	2.88	10.97	<12	2.22	18.28	3.72	4.79	<1.5		18.93	4.13
Endo-PSH SRB, aerial part	<24.50	24.63	<49	<1.5	3,67	3.94	2.91	2.58	10.60	<12	2.70	45.15	10.84	5.16	<1.5		58.57	3.22

Values with “<“ indicate concentrations below the LoQ (limit of quantification), but above the LoD (limit of detection), where the compound was detected, but not quantifiable. Abbreviations: HP—hydropriming; SRB—Serbian strain; ITA—Italian strain, n.a—not analyzed.

## Data Availability

The data that support the findings of this study are available within the manuscript and its Supplementary Material.

## Supplementary Materials

The following supporting information can be downloaded at https://www.mdpi.com/article/10.3390/antiox15091145/s1. Figure S1: ITS sequence of tested *S. commune* strains (FBA535—SRB strain, FBA538—ITA strain); Figure S2: Phylogenetic tree of *S. commune* strains (FBA535—SRB strain, FBA538—ITA strain); Figure S3: Congo red test of endo- and exo-PSHs from ITA and SRB *S. commune* strains; Figure S4: ^1^H NMR spectra of (a) endo-PSH SRB, (b) exo-PSH SRB, (c) endo-PSH ITA, and (d) exo-PSH ITA in DMSO. Abbreviations: endo-PSH—endopolysaccharide; exo-PSH—exopolysaccharide, SRB—Serbian strain, ITA—Italian strain; Figure S5: ^13^C NMR spectrum of endo-PSHs SRB in DMSO. Abbreviations: endo-PSH—endopolysaccharide; exo-PSH—exopolysaccharide, SRB—Serbian strain, ITA—Italian strain; Figure S6: ^1^H NMR spectra of enzymatically treated (a) exo-PSH SRB, (b) exo-PSH ITA, and (c) endo-PSH ITA in DMSO; Figure S7: ATR-FTIR spectrum of absolute ethanol; Figure S8: Chlorophyll content in *Pisum sativum* L. methanolic seedling extracts after biopriming with fungal polysaccharides under (a) optimal and (b) drought conditions. Abbreviations: HP—hydropriming; endo-PSH—endopolysaccharide; exo-PSH—exopolysaccharide, SRB—Serbian strain, ITA—Italian strain; Figure S9: Hydroxyl radical (OH)-scavenging capacity and lipid peroxidation (LP) inhibition in *Pisum sativum* L. extracts after biopriming with fungal polysaccharides under (a) optimal and (b) drought conditions; Figure S10: ABTS and DPPH radical-scavenging activity and FRAP reduction potential of *Pisum sativum* L. methanolic seedling extracts after biopriming with fungal polysaccharides under (a) optimal and (b) drought conditions. Abbreviations: TE—trolox equivalent, AAE—ascorbic acid equivalent, HP—hydropriming; SRB—Serbian strain, ITA—Italian strain, endo-PSH—endopolysaccharide; exo-PSH—exopolysaccharide, AP—aerial part); Figure S11: Pearson analysis of enzymatic and non-enzymatic antioxidant activity in (a) optimal and (b) drought conditions. Abbreviations: TPC—total phenolic content; TFC—total flavonoid content, LP—lipid peroxidation, POD—pyrogallol peroxidase, GPX—guaiacol peroxidase, APX—ascorbate peroxidase, CAT—catalase, TPR—total protein content, PRO—proline; Figure S12: PCA and loading plots for enzymatic and non-enzymatic antioxidant activity in (a) optimal and (b) drought conditions. Abbreviations: HP—hydropriming; SRB—Serbian strain; ITA—Italian strain; AE—aerial part, LP—lipid peroxidation, PRO—proline, PSH—polysaccharide; TPC—total phenolic content; TFC—total flavonoid content, POD—pyrogallol peroxidase, GPX—guaiacol peroxidase, APX—ascorbate peroxidase; Table S1. Compounds (38) from standard Mix1 and their retention times and MRM parameters; Table S2. Compounds (24) from standard Mix2 and their retention times and MRM parameters; Table S3: Total phenolic (TPC) and total flavonoid content (TFC) in extracts of *P. sativum* L. after biopriming with fungal polysaccharides under optimal and drought conditions.

## Abbreviations

The following abbreviations are used in this manuscript.

AAE	ascorbic acid equivalent
ABTS	2,2-azino-bis(3-ethylbenzothiazoline-6-sulfonic acid)
APX	ascorbate peroxidase
CAT	catalase
dH_2_O	distilled water
DMSO	dimethyl sulfoxide
DPPH	2,2-diphenyl-1-picrylhydrazyl radical
Endo-PSH	endopolysaccharide
Exo-PSH	exopolysaccharide
FC	Folin–Ciocâlteu
FRAP	ferric reducing antioxidant power
FTIR	Fourier-transform infrared spectroscopy
GAE	gallic acid equivalent
GPX	guaiacol peroxidase
Hetero-PSH	heteropolysaccharides
HP	hydropriming
ITA	Italian strain
LC–MS/MS	liquid chromatography coupled with tandem mass-spectrometric detection
MeOH	methanolic extract prepared with 80% methanol
NMR	nuclear magnetic resonance
OH	hydroxyl radical
PCA	principal component analysis
POD	pyrogallol peroxidase
PRO	proline
PSH	polysaccharide
QE	quercetin equivalent
ROS	reactive oxygen species
RT	room temperature
SEM	scanning electron microscopy
SRB	Serbian strain
TE	trolox equivalent
TFC	total flavonoid content
TGA–DTA	thermogravimetric and differential thermal analysis
TPC	total phenolic content
TPR	total protein content
