# Supplementary material for: Biopriming with Schizophyllum commune Polysaccharides Modulates Antioxidant and Biochemical Responses in Pisum sativum L. Seedlings
Source: Antioxidants (Basel). 2026 Sep 9;15(9):1145. doi: 10.3390/antiox15091145 (PMC13603578; doi:10.3390/antiox15091145)
Supplement: Supplementary file 1 [file antioxidants-15-01145-s001.zip › antioxidants-4528555-supplementary.pdf]

antioxidants

>FBA535 SRB

**TTCTGATCCTGTGCACCTTATGTAGTCCCAAAGCCTTCACGGGCGGCGGTTGA
CTACGTCTACCTCACACCTTAAAGTATGTTAACGAATGTAATCATGGTCTTGACAGACCCTAAAAA
GTTAATACAACTTTCGACAACGGATCTCTTGGCTCTCGCATCGATGAAGAACGCAGCGAAATGCG
ATAAGTAATGTGAATTGCAGAATTCAGTGAATCATCGAATCTTTGAACGCACCTTGCGCCCTTTGG
TATTCCGAGGGGCATGCCTGTTTGAGTGTCATTAAATACCATCAACCCTCTTTTGACTTCGGTCTC
GAGAGTGGCTTGGAAGTGGAGGTCTGCTGGAGCCTAACGGAGCCAGCTCCTCTTAAATGTATTAG
CGGATTTCCCTTGCGGGATCGCGTCTCCGATGTGATAATTTCTACGTCGTTGACCATCTCGGGGCT
GACCTAGTCAGTTTCAATAGGAGTCTGCTTCTAACCGTCTCTTGACCGAGACTAGCGACTTGTGC
GCTAACT TTTGACTTGACCTCAAATCAGGTAGGACTACCCGCTGAACTTAAGCATATC**

>FBA538 ITA

**CCTGTGCACCTTATGTAGTCCCAAAGCCTTCACGGGCGGCGGTTGACTACGTC
TACCTCACACCTTAAAGTATGTTAACGAATGTAATCATGGTCTTGACAGACCCTAAAAAGTTAATAC
AACTTTCGACAACGGATCTCTTGGCTCTCGCATCGATGAAGAACGCAGCGAAATGCGATAAGTAA
TGTGAATTGCAGAATTCAGTGAATCATCGAATCTTTGAACGCACCTTGCGCCCTTTGGTATTCCGA
GGGGCATGCCTGTTTGAGTGTCATTAAATACCATCAACCCTCTTTTGACTTCGGTCTCGAGAGTGG
CTTGGAAGTGGAGGTCTGCTGGAGCCTAACGGAGCCAGCTCCTCTTAAATGTATTAGCGGATTTC
CCTTGCGGGATCGCGTCTCCGATGTGATAATTTCTACGTCGTTGACCATCTCGGGGCTGACCTAGT
CAGTTTCAATAGGAGTCTGCTTCTAACCGTCTCTTGACCGAGACTAGCGACTTGTGCGCTAACTTT
TGACTTGACCTCAAATCAGGTAGGACTACCCGCTGAACTTAAGCATATC**

Figure S1. ITS sequence of tested *S. commune* strains (FBA535 - SRB strain and FBA538 - ITA strain).

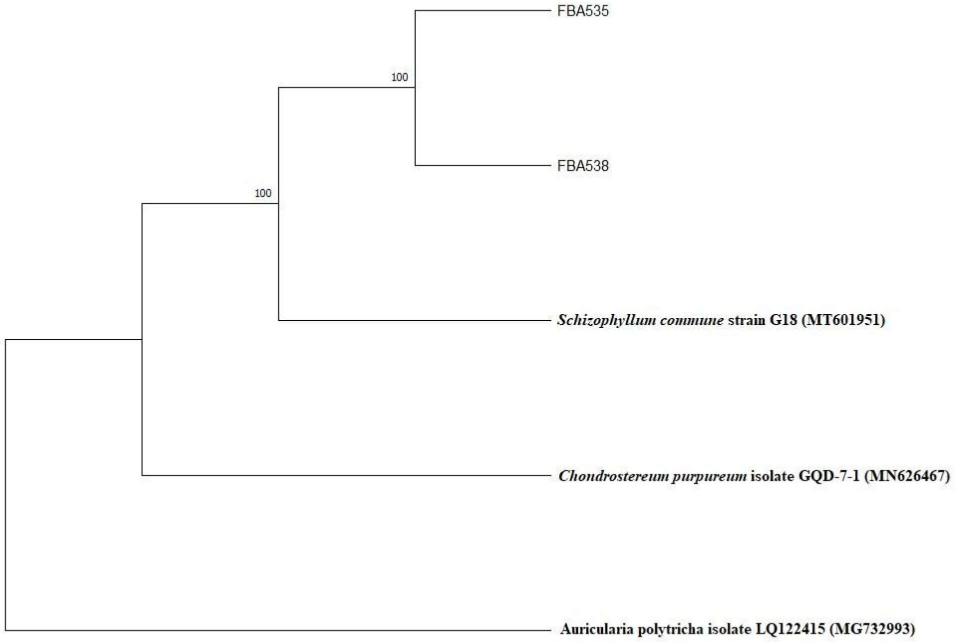

Figure S2. Phylogenetic tree of *S. commune* strains (FBA535 – SRB strain, FBA538 – ITA strain).

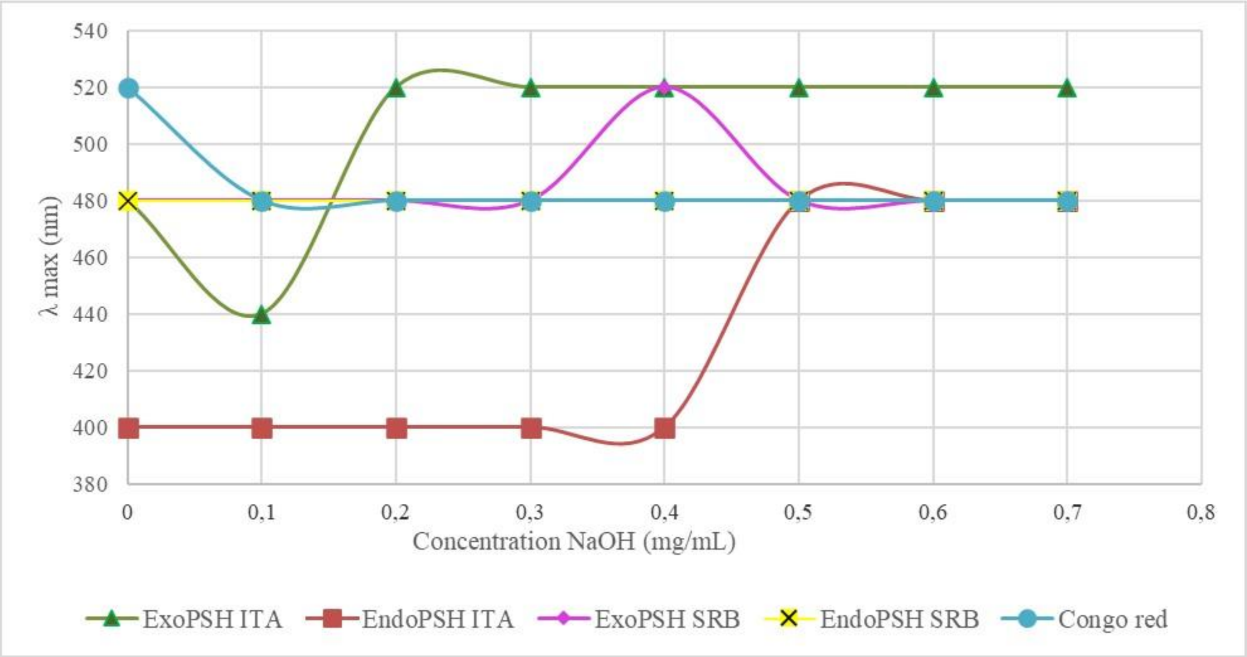

Figure S3. Congo red test of endo- and exo-PSHs from ITA and SRB *S. commune* strains.

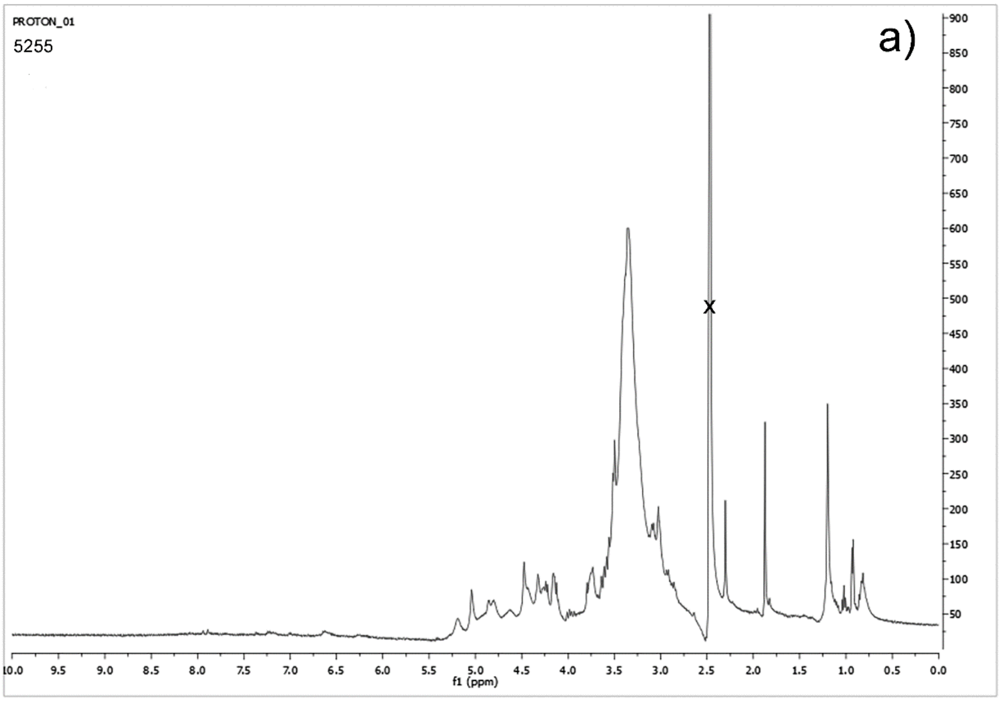

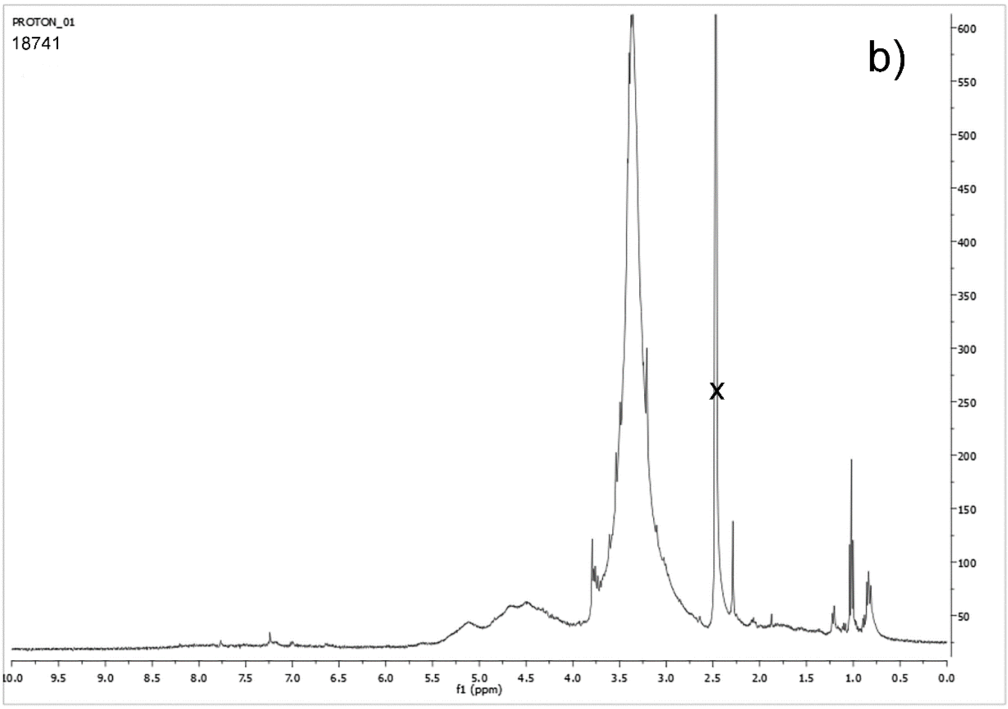
PROTON_01
18741
b)
x
f1 (ppm)

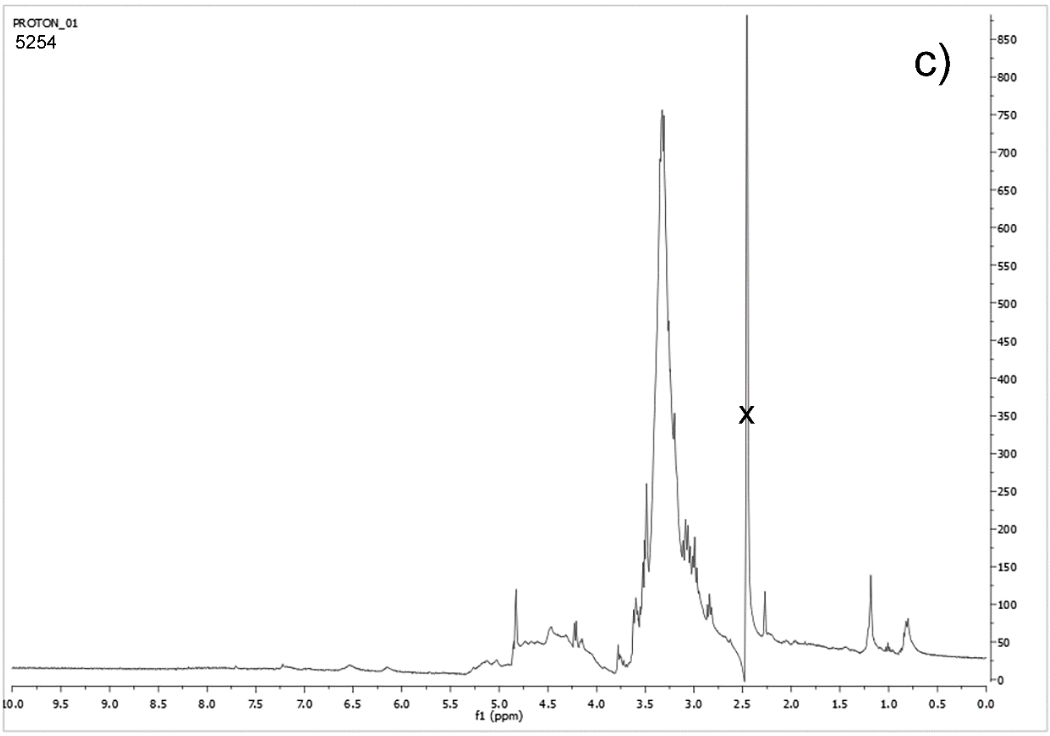
PROTON_01
5254
c)
x
f1 (ppm)

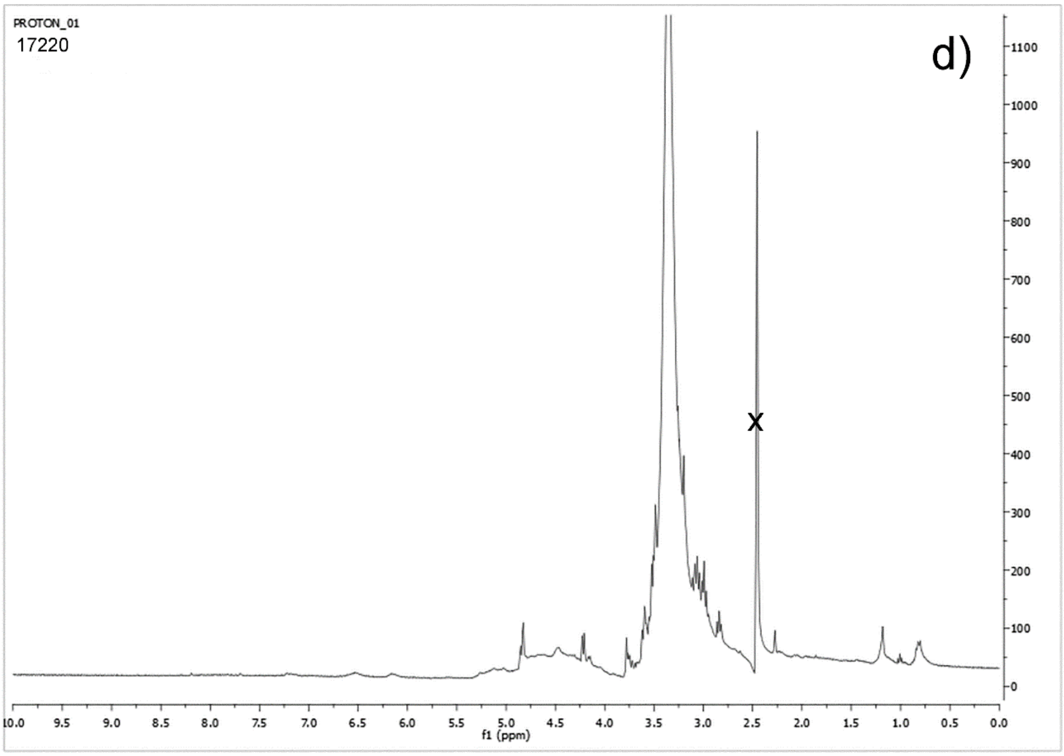

Figure S4. ^{1}H NMR spectra of a) Endo-PSHs SRB b) Exo-PSHs SRB c) Endo-PSHs ITA d) Exo-PSHs ITA in DMSO. Abbreviations: Endo-PSH – endopolysaccharide; Exo-PSH – exopolysaccharide, SRB – Serbian strain, ITA – Italian strain.

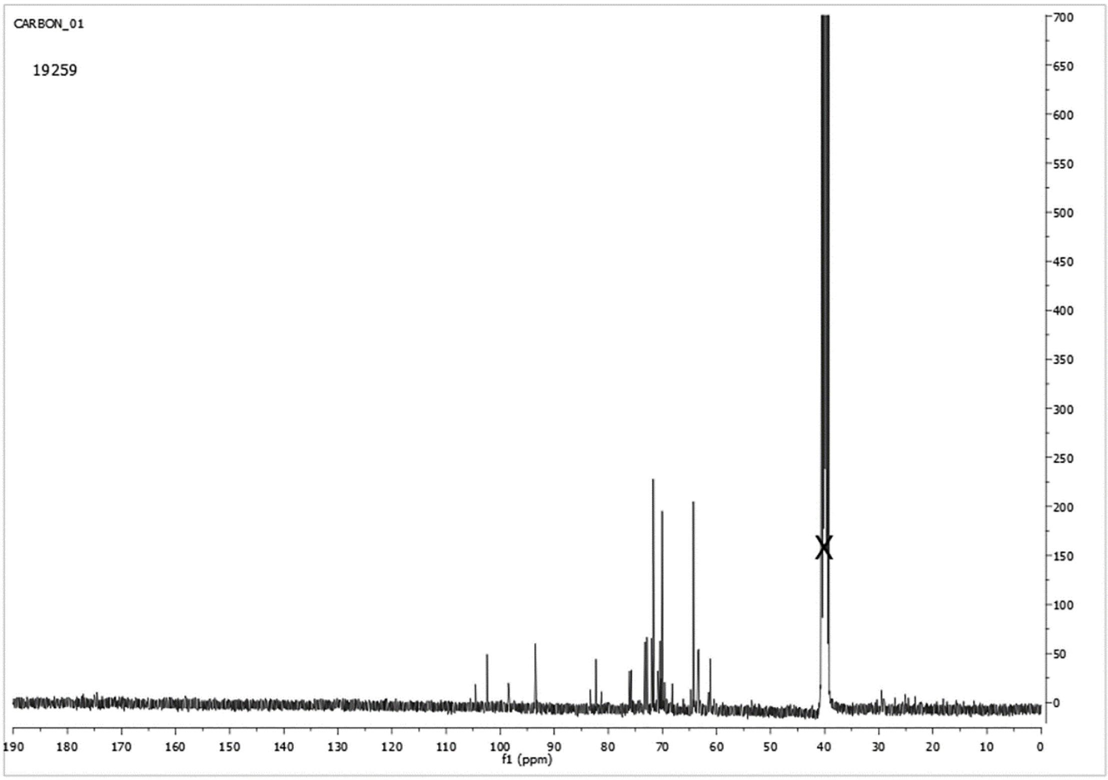

Figure S5. ^{13}C NMR spectrum of Endo-PSHs SRB in DMSO.

https://doi.org/10.3390/antiox15091145

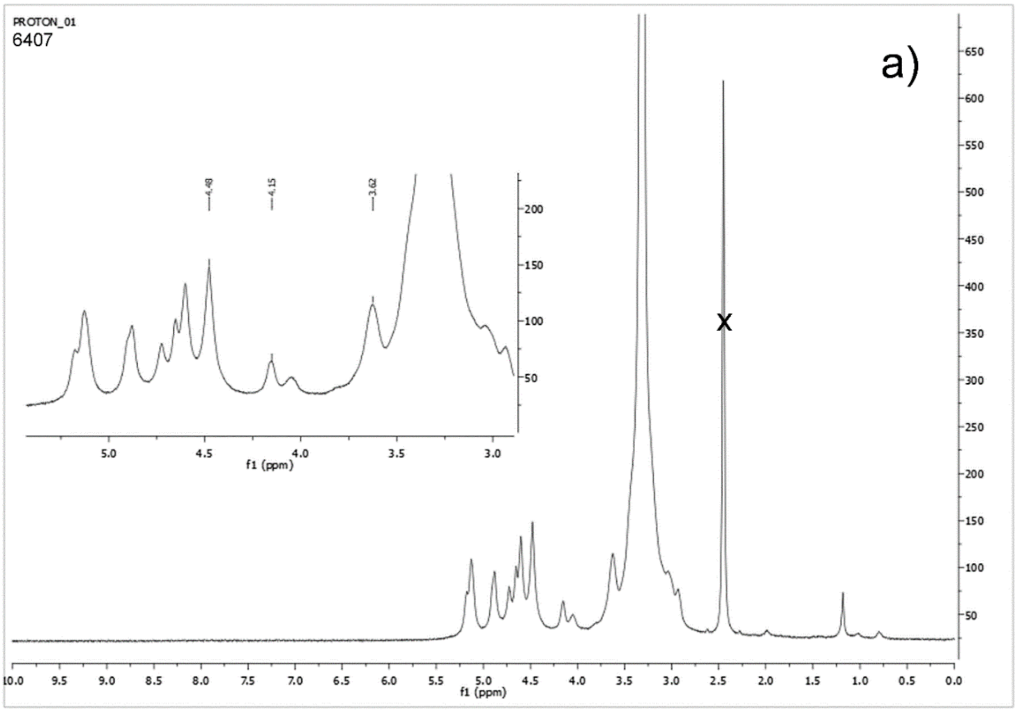
PROTON_01
6407
a)
4.48
4.15
3.62
f1 (ppm)
x
f1 (ppm)

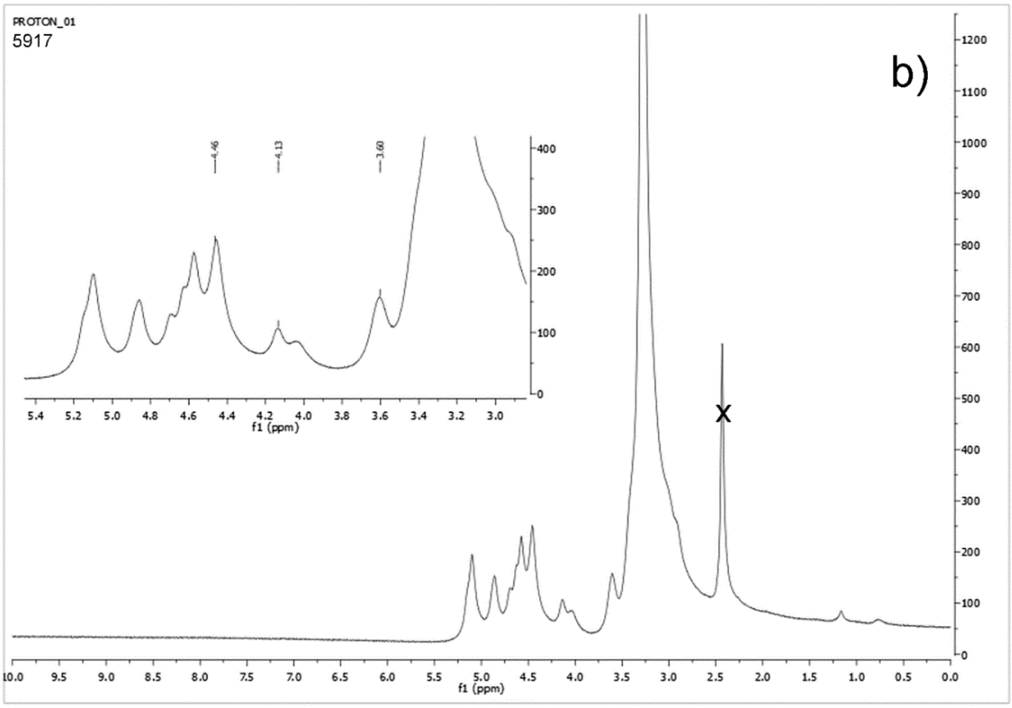
PROTON_01
5917
b)
4.46
4.13
3.60
f1 (ppm)
x
f1 (ppm)

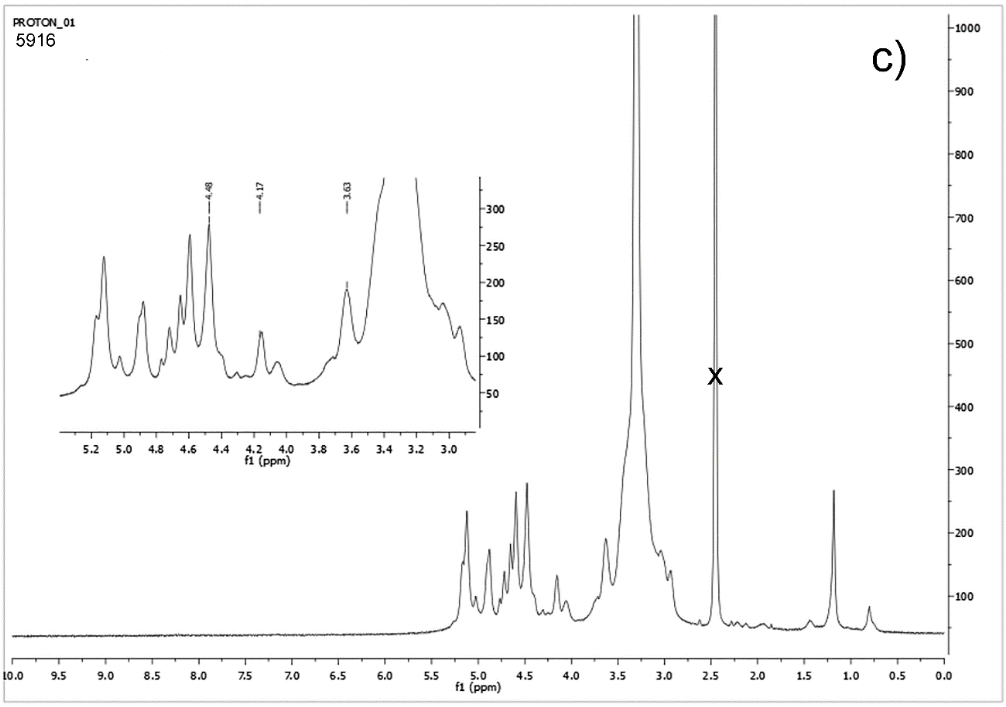

Figure S6. ^{1}H NMR spectra of enzymatically treated a) Exo-PSHs SRB b) Exo-PSHs ITA c) Endo-PSHs ITA in DMSO. Abbreviations: Endo-PSH – endopolysaccharide; Exo-PSH – exopolysaccharide, SRB – Serbian strain, ITA – Italian strain.

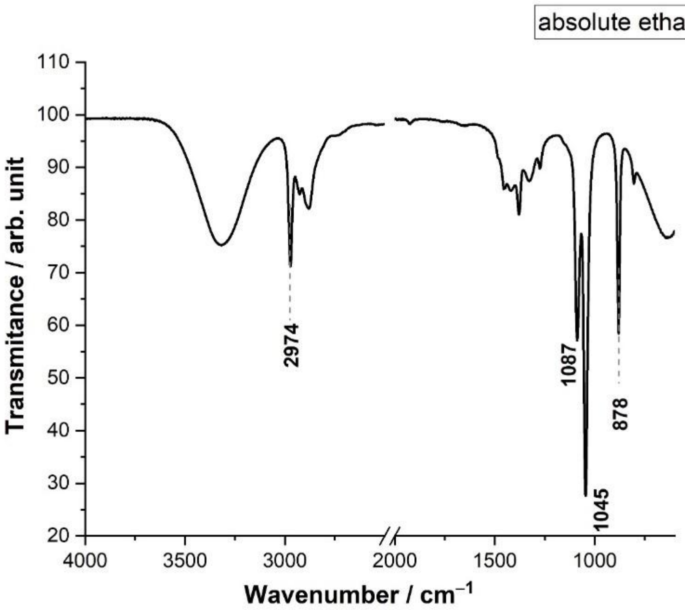

Figure S7. ATR-FTIR spectrum of absolute ethanol.

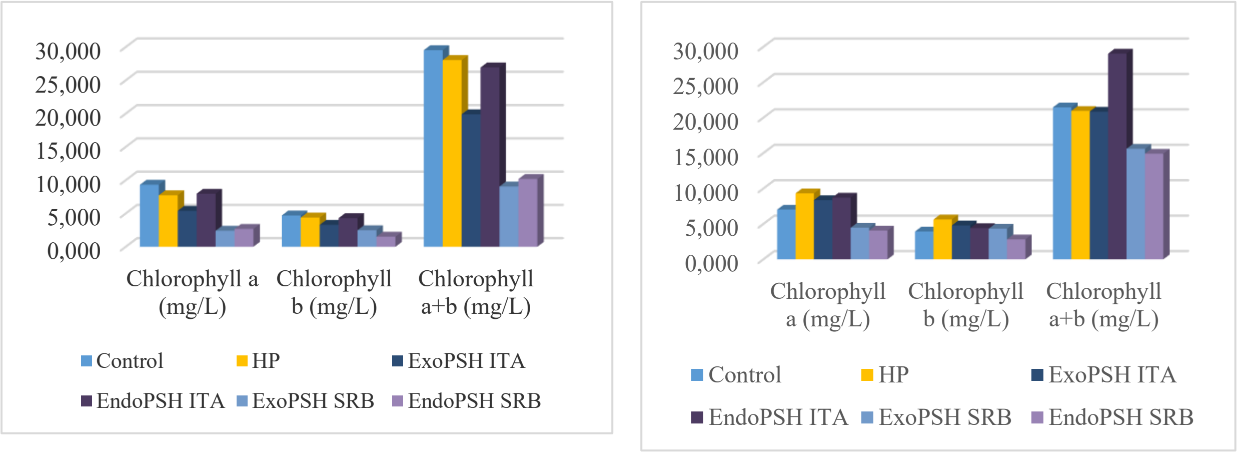

a) b)

Figure S8. Chlorophyll content in *Pisum sativum* L. methanolic seedling extracts after biopriming with fungal polysaccharides under a) optimal and b) drought conditions. Abbreviations: HP – hydropriming; Endo-PSH – endopolysaccharide; Exo-PSH – exopolysaccharide, SRB – Serbian strain, ITA -Italian strain.

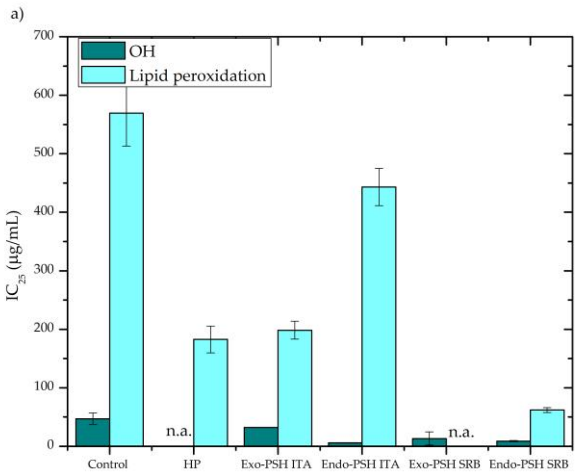

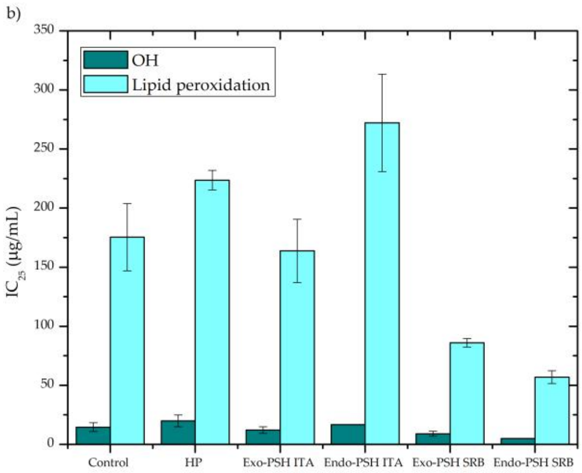

Figure S9. Hydroxyl radical (OH) scavenging capacity and lipid peroxidation (LP) inhibition in *Pisum sativum* L. extracts after biopriming with fungal polysaccharides under (a) optimal and (b) drought conditions.

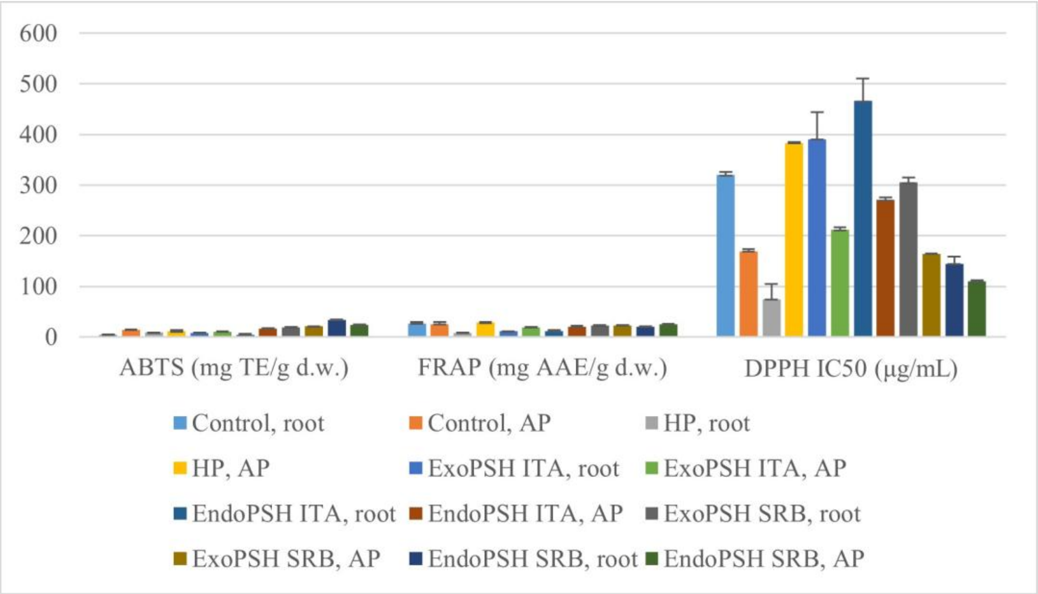

a)

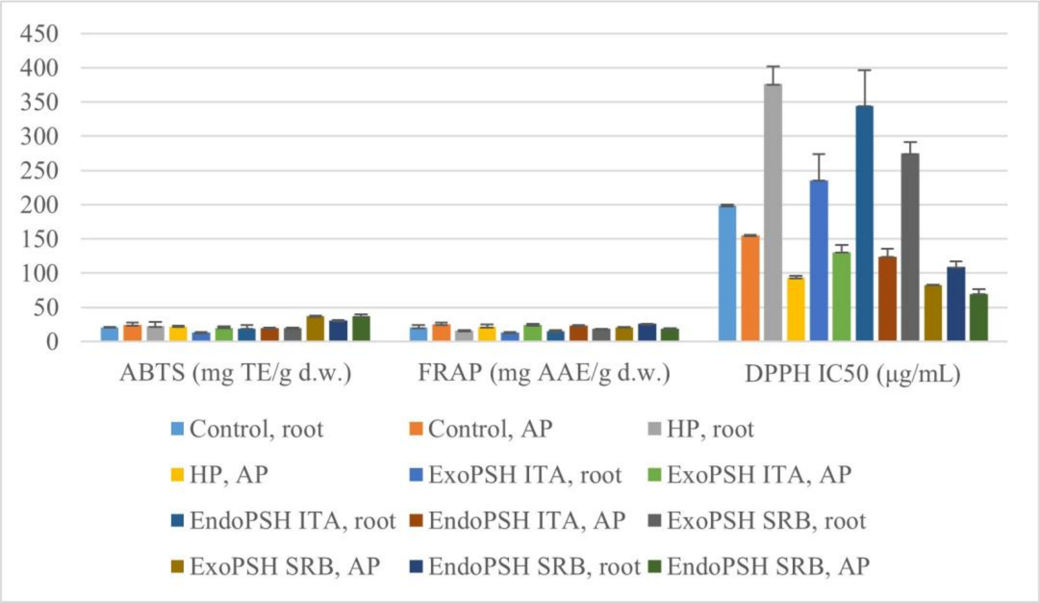

b)

Figure S10. ABTS and DPPH radical scavenging activity and FRAP reduction potential of *Pisum sativum* L. methanolic seedling extracts after biopriming with fungal polysaccharides under (a) optimal and (b) drought conditions. Abbreviations: TE – Trolox equivalent, AAE – ascorbic acid equivalent, HP – hydropriming; SRB – Serbian strain, ITA – Italian strain, Endo-PSH – endopolysaccharide; Exo-PSH – exopolysaccharide, AP – aerial part).

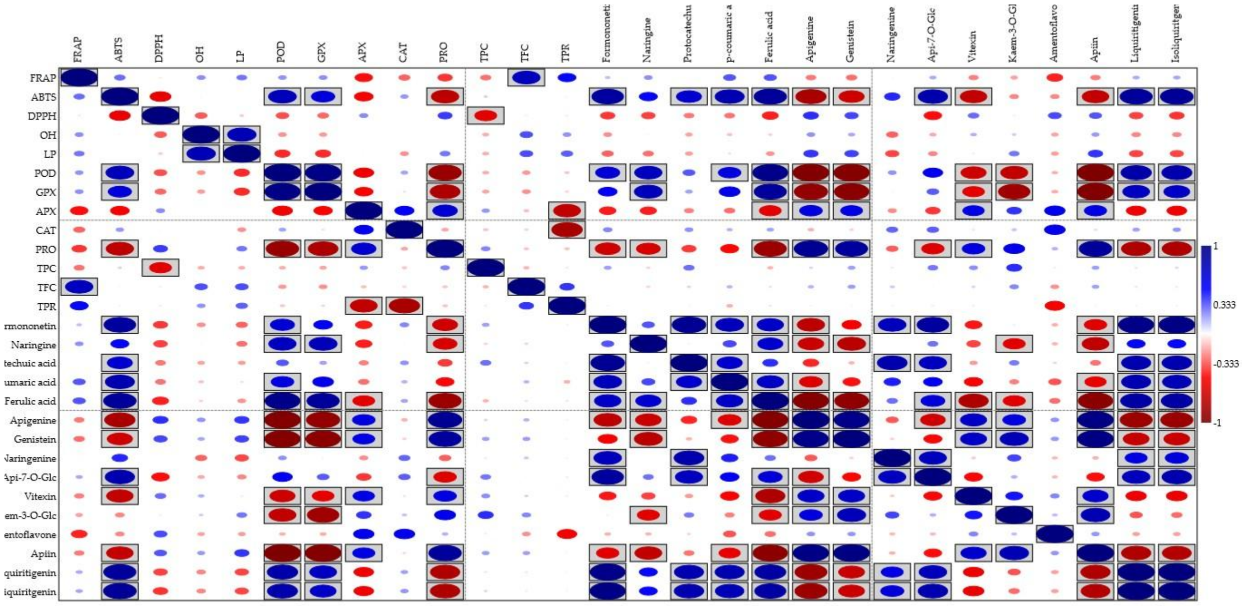

a)

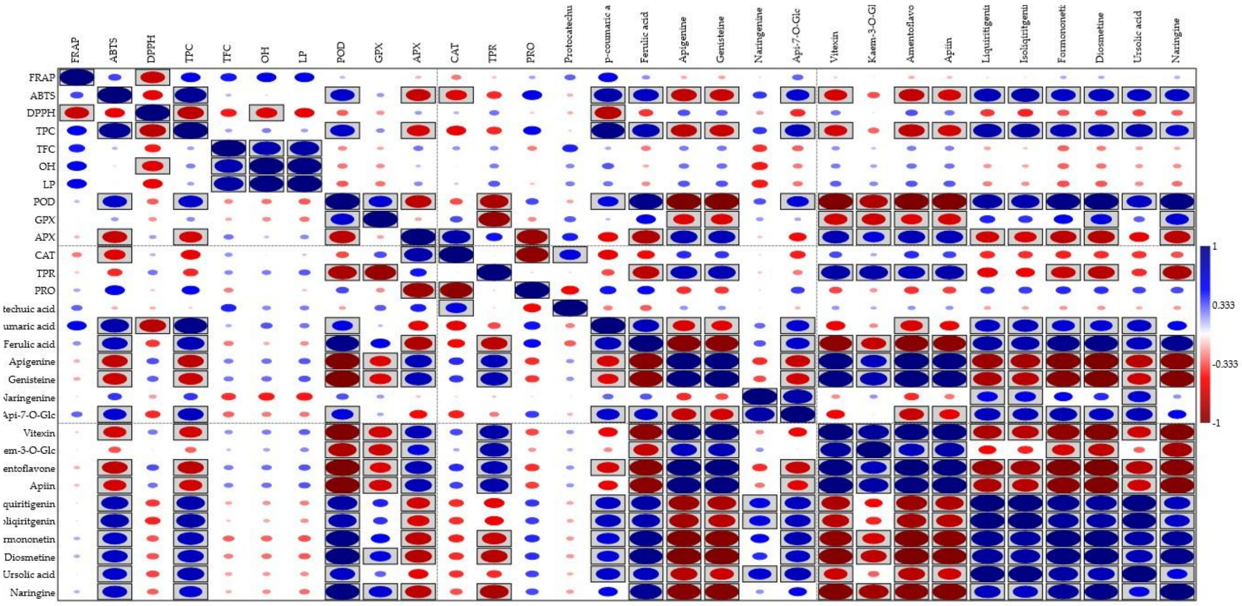

b)

Figure S11. Pearson analysis for enzymatic and non-enzymatic antioxidant activity in (a) optimal and (b) drought conditions. Abbreviations: TPC – Total phenolic content; TFC – Total flavonoid content, LP – lipid peroxidation, POD – pyrogallol peroxidase, GPX – guaiacol peroxidase, APX – ascorbate peroxidase, CAT – catalase, TPR – Total protein content, PRO – proline.

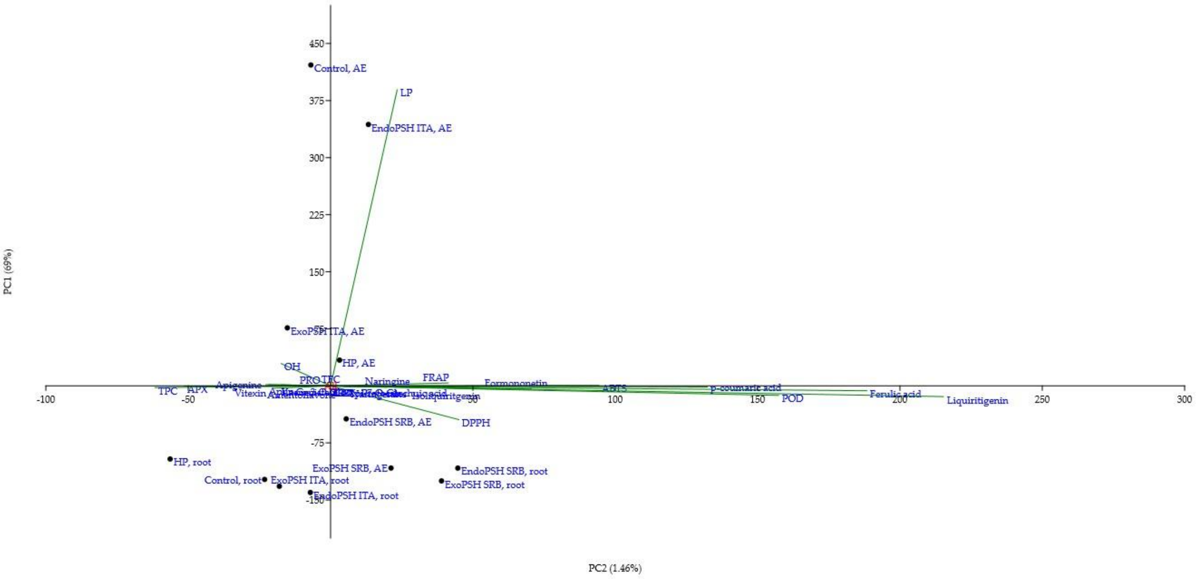

https://doi.org/10.3390/antiox15091145

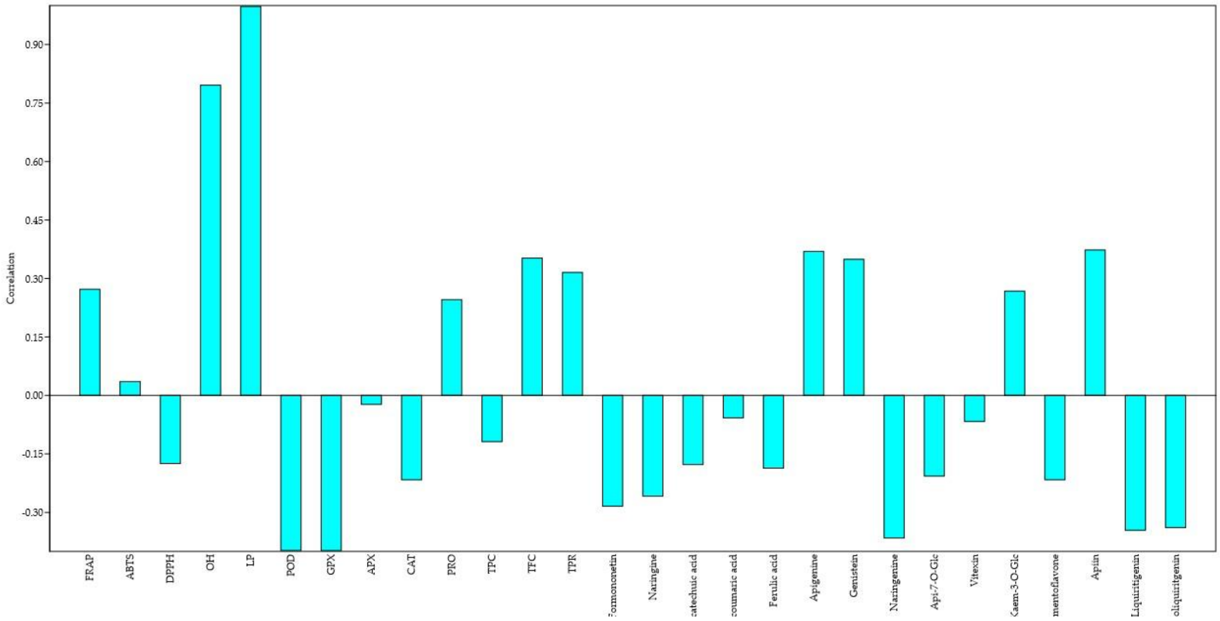
Correlation
0.90
0.75
0.60
0.45
0.30
0.15
0.00
-0.15
-0.30
FRAP
ABTS
DPPH
OH
LP
POD
GPX
APX
CAT
PRO
TPC
TFC
TPR
Naringine
Ferulic acid
Apigenine
Genistein
Naringenine
Api-7-O-Glc
Vitexin
Apiin
Liquiritigenin

a)

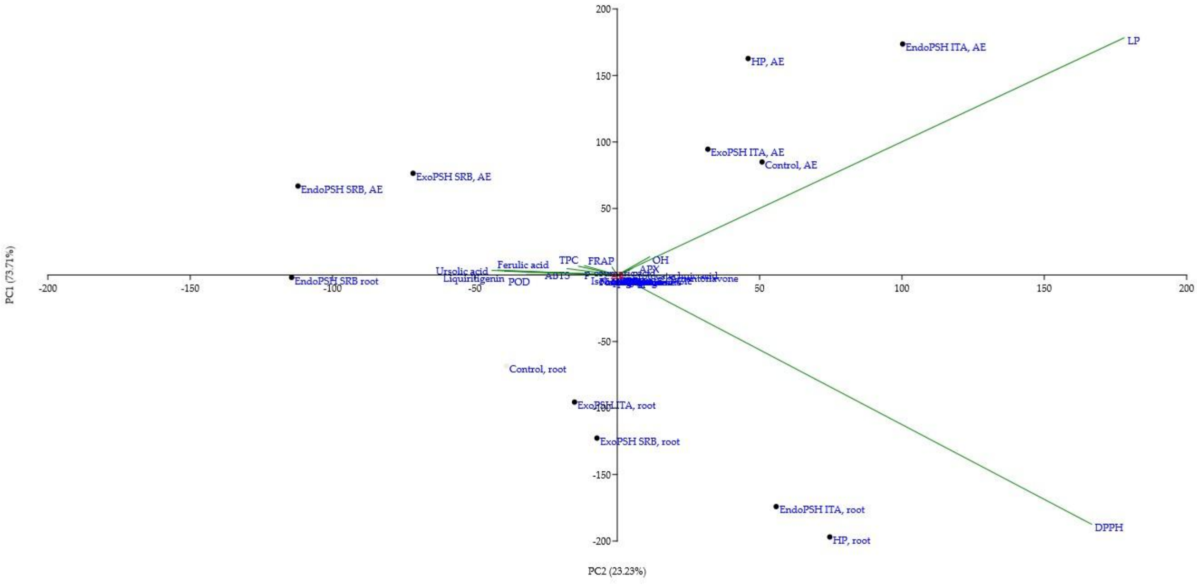
PC1 (73.71%)
PC2 (23.23%)
200
150
100
50
-50
-150
-200
-100
0
LP
DPPH
OH
FRAP
TPC
APX
Ferulic acid
Ursolic acid
Liquiritigenin
POD
ABTS
EndoPSH ITA, AE
HP, AE
ExoPSH ITA, AE
Control, AE
EndoPSH SRB, AE
ExoPSH SRB, AE
EndoPSH SRB root
Control, root
ExoPSH SRB, root
EndoPSH ITA, root
HP, root

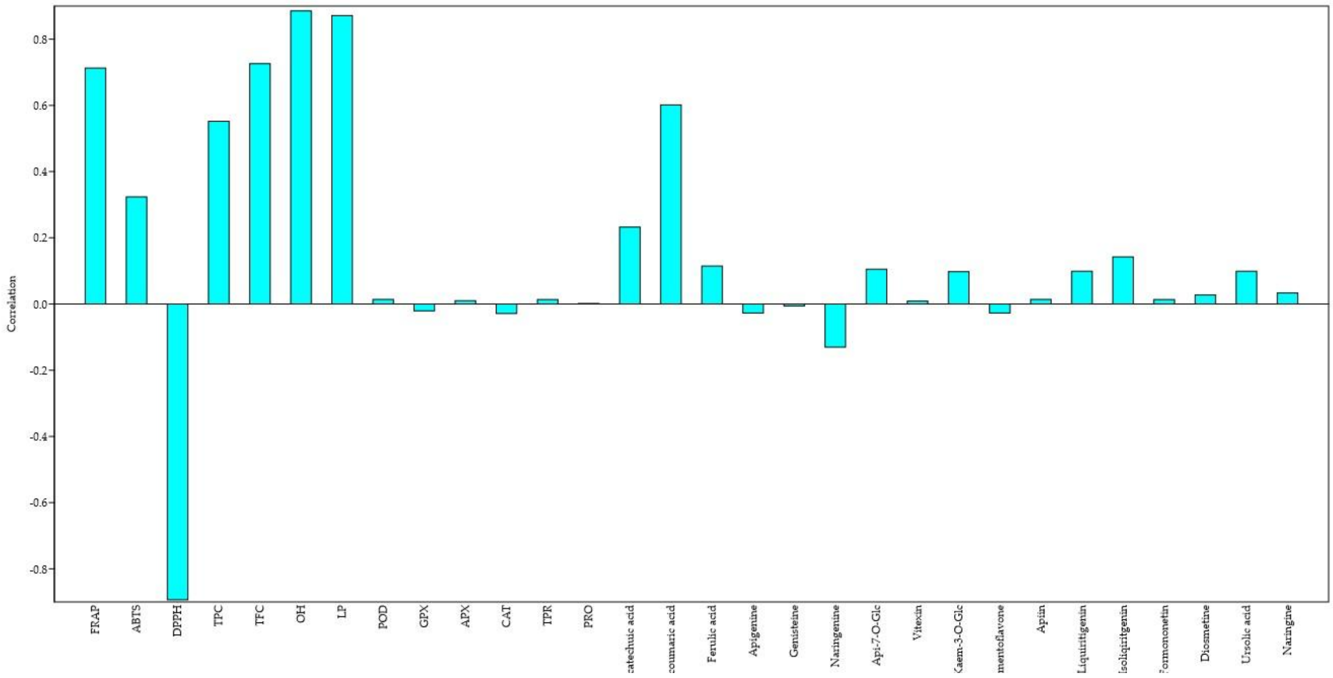

b)

Figure S12. PCA and loading plots for enzymatic and non-enzymatic antioxidant activity in (a) optimal and (b) drought conditions. Abbreviations: HP – hydropriming; SRB – Serbian strain; ITA – Italian strain; AE – aerial part, LP – lipid peroxidation, PRO – proline, PSH – Polysaccharide; TPC – Total phenolic content; TFC – Total flavonoid content, POD – pyrogallol peroxidase, GPX – guaiacol peroxidase, APX – ascorbate peroxidase.

Table S1. 38 compounds from standard Mix1 and their retention times and MRM parameters.

Compound	tR [min]	m/z precursor	m/z product	Vcollision [V]	Vfragmentor [V]
p-OH Benzoic acid	1.2	137	93	10	80
Cinnamic acid	4.0	147	103	5	100
Protocatechuic acid	0.9	153	109	9	105
Gentisic acid	1.0	153	109	9	100
p-Coumaric acid	1.8	163	119	9	90
o-Coumaric acid	2.8	163	119	5	100
Umbelliferon	1.9	161	133	19	120
Esculetin	1.2	177	133	15	105
Scopoletin	1.8	191	176	8	80
Ferulic acid	2.0	193	134	11	90
Syringic acid	1.4	197	182	7	90
3,4-Dimethoxycinnamic acid	3.1	207	103	7	110
Sinapic acid	2.0	223	193	17	100
Daidzein	3.6	253	208	31	145
Genistein	4.3	269	133	32	145

https://doi.org/10.3390/antiox15091145

Apigenin	4.8	269	117	25	130
Naringenin	4.0	271	151	16	130
Luteolin	4.2	285	133	25	135
Kaempferol	4.7	285	285	0	130
Epicatechin	1.1	289	245	10	150
Chrysoeryol	4.9	299	284	20	125
Quercetin	3.9	301	151	15	130
Isorhamnetin	4.9	315	300	21	160
Myricetin	2.9	317	179	20	150
Chlorogenic acid	0.9	353	191	10	100
Matairesinol	3.7	357	122	24	130
Secoisolariciresinol	2.9	361	165	26	130
Apigenin-7-O-Glc	2.8	431	268	41	135
Vitexin	2.0	431	311	22	200
Baicalin	3.3	455	269	22	140
Kaempferol-3-O-Glc	3.0	447	284	30	190
Luteolin-7-O-Glc	2.3	447	285	30	230
Quercitrin	3.0	447	300	27	190
Quercetin-3-O-Glc	2.4	463	300	30	210
Quercetin-3-O-Gal	2.4	463	300	30	200
Amentoflavone	5.9	537	269	35	220
Apiin	2.7	563	269	36	250
Rutin	2.4	609	300	42	135

Table S2. 24 compounds from standard Mix2 and their retention times and MRM parameters

Compound	tR [min]	m/z precursor	m/z product	Vcollision [V]	Vfragmentor [V]
Hydroxyphenylacetic acid	1.15	151	107	10	80
Isoscopoletin	1.58	191	176	5	120
Resveratrol	2.39	227	185	15	130
Alizarin	5.50	239	210	30	190
Dantron	7.43	239	211	25	200
Chrysin	6.04	253	143	23	150
Pinocembrin	5.78	255	213	18	130
Liquiritigenin	3.05	255	119	22	100
Isoliquiritigenin	4.63	255	119	22	100
Abscisic acid	3.50	263	153	0	170
Formononetin	5.18	267	252	16	100
Aloe-emodin	5.83	269	240	19	170
Emodin	7.67	269	225	25	160
Pinostrobin	6.27	269	269	0	130
Galangin	6.27	269	169	23	150
Rhein	6.48	283	239	10	90
Diosmetin	4.74	299	284	20	140
Hesperetin	4.14	301	164	23	125
Ellagic acid	2.38	301	301	0	152
Rhamnetin	5.40	315	165	19	140
Ursolic acid	9.15	455	455	12	300
Glycyrrhizic acid	8.67	469	425	40	280

Naringin	2.25	579	271	35	230
Glycyrrhizin	6.98	821	351	45	220

Table S3. Total phenolic (TPC) and total flavonoid content (TFC) in extracts of *P. sativum* L. after biopriming with fungal polysaccharides under optimal and drought conditions.

Treatment	TPC (mg GAE/g d.w.)	TFC (mg QE/g d.w.)
	Optimal conditions	
Control, root	21.47 ± 3.75^{a,d}	2.21 ± 0.11^{a,d}
Control, aerial part	20.69 ± 2.82^{a,d}	1.40 ± 0.26^{a,d}
HP, root	45.79 ± 3.45^{a,d}	0.12 ± 0.05^{b,d}
HP, aerial part	25.09 ± 0.88^{a,d}	1.11 ± 0.12^{b,d}
Exo-PSH ITA, root	20.65 ± 1.20^{c,d}	0.55 ± 0.05^{a,d}
Exo-PSH ITA, aerial part	18.20 ± 1.18^{c,d}	1.37 ± 0.09^{a,d}
Endo-PSH ITA, root	12.05 ± 1.55^{a,d}	0.17 ± 0.06^{a,d}
Endo-PSH ITA, aerial part	17.13 ± 1.13^{a,d}	1.15 ± 0.33^{a,d}
Exo-PSH SRB, root	10.82 ± 0.26^{a,d}	0.54 ± 0.00^{a,d}
Exo-PSH SRB, aerial part	18.60 ± 0.84^{a,d}	0.85 ± 0.00^{a,d}
Endo-PSH SRB, root	30.41 ± 0.35^{b,d}	1.12 ± 0.25^{a,d}
Endo-PSH SRB, aerial part	24.17 ± 0.02^{b,d}	0.82 ± 0.19^{a,d}
	Drought conditions	
Control, root	12.70 ± 0.71^{a,e}	0.73 ± 0.17^{a,d}
Control, aerial part	18.82 ± 0.68^{a,e}	1.32 ± 0.13^{a,d}
HP, root	11.36 ± 0.76^{a,e}	0.74 ± 0.05^{b,d}
HP, aerial part	17.43 ± 0.85^{a,e}	0.11 ± 0.01^{b,d}
Exo-PSHs ITA, root	8.55 ± 0.33^{b,e}	0.30 ± 0.04^{a,d}
Exo-PSHs ITA, aerial part	14.94 ± 0.39^{a,e}	1.90 ± 0.52^{a,d}
Endo-PSHs ITA, root	10.13 ± 0.18^{a,e}	1.02 ± 0.03^{a,d}
Endo-PSHs ITA, aerial part	16.06 ± 1.41^{a,e}	2.09 ± 0.34^{a,d}
Exo-PSHs SRB, root	15.99 ± 0.86^{a,e}	0.43 ± 0.09^{a,d}

https://doi.org/10.3390/antiox15091145

Exo-PSHs SRB, aerial part	23.03 ± 0.51^{a,e}	1.62 ± 0.15^{a,d}
Endo-PSHs SRB, root	23.28 ± 0.50^{a,e}	0.38 ± 0.04^{a,d}
Endo-PSHs SRB, aerial part	28.56 ± 1.22^{a,e}	1.03 ± 0.11^{a,d}

The results are expressed as the mean ± standard deviation (SD) from three replicates. Statistical differences among treatments were evaluated using Two-way ANOVA followed by Tukey's HSD post hoc test ($p < 0.05$). Superscript letters (a-c) within each column denote significant differences regarding treatment, while distinct superscript letters (d and e) denote significant differences regarding condition (optimal and drought). Abbreviations: HP – hydropriming; SRB – Serbian strain; ITA – Italian strain; PSH – Polysaccharide; TPC – Total phenolic content; GAE – Gallic acid equivalents; TFC – Total flavonoid content; QE - Quercetin equivalents; d.w. – dry weight.
